# Online 2D Fluorescence Monitoring in Microtiter Plates Allows Prediction of Cultivation Parameters and Considerable Reduction in Sampling Efforts for Parallel Cultivations of *Hansenula polymorpha*

**DOI:** 10.3390/bioengineering9090438

**Published:** 2022-09-04

**Authors:** Christoph Berg, Nina Ihling, Maurice Finger, Olivier Paquet-Durand, Bernd Hitzmann, Jochen Büchs

**Affiliations:** 1 AVT—Aachener Verfahrenstechnik, Biochemical Engineering, RWTH Aachen University, Forckenbeckstraße 51, 52074 Aachen, Germany; 2 Department of Process Analytics & Cereal Science, Institute for Food Science and Biotechnology, University of Hohenheim, Garbenstraße 23, 70599 Stuttgart, Germany

**Keywords:** 2D fluorescence spectroscopy, online monitoring, multivariate data analysis, high-throughput, microtiter plate

## Abstract

Multi-wavelength (2D) fluorescence spectroscopy represents an important step towards exploiting the monitoring potential of microtiter plates (MTPs) during early-stage bioprocess development. In combination with multivariate data analysis (MVDA), important process information can be obtained, while repetitive, cost-intensive sample analytics can be reduced. This study provides a comprehensive experimental dataset of online and offline measurements for batch cultures of *Hansenula polymorpha*. In the first step, principal component analysis (PCA) was used to assess spectral data quality. Secondly, partial least-squares (PLS) regression models were generated, based on spectral data of two cultivation conditions and offline samples for glycerol, cell dry weight, and pH value. Thereby, the time-wise resolution increased 12-fold compared to the offline sampling interval of 6 h. The PLS models were validated using offline samples of a shorter sampling interval. Very good model transferability was shown during the PLS model application to the spectral data of cultures with six varying initial cultivation conditions. For all the predicted variables, a relative root-mean-square error (RMSE) below 6% was obtained. Based on the findings, the initial experimental strategy was re-evaluated and a more practical approach with minimised sampling effort and elevated experimental throughput was proposed. In conclusion, the study underlines the high potential of multi-wavelength (2D) fluorescence spectroscopy and provides an evaluation workflow for PLS modelling in microtiter plates.

## 1. Introduction

Over the last decades, various microbioreactor designs, including stirred [1,2,3,4], shaken [5,6,7], sparged [8,9,10] and pumped systems [11,12] have been developed to accelerate early up-stream bioprocess development [13,14]. To follow the Process Analytical Technology (PAT) guidance for process analytics and control [15], these microscale cultivation vessels are often equipped with a miniaturised periphery [16], enabling at-line and online process monitoring down to droplet-size cultivation volumes [17,18]. Amongst others, spectroscopic measurement methods based on Raman [19,20], impedance [21,22,23] or single-wavelength fluorescence [24,25] have successfully been transferred from lab-scale reactors to microscale cultivation systems.

For exploring and exploiting the information of the acquired spectral datasets, multivariate data analysis methods (MVDA) or chemometrics are usually applied [26,27]. For example, principal component analysis (PCA) can be used to condense data with high dimensionality to only a few uncorrelated dimensions. The dimensional reduction is conducted by transferring the spectral data into a new coordinate system of so-called principal components (PCs), which are iteratively generated according to the maximum residing variance within the spectral data [28,29,30]. In bioprocess engineering, PCA has, for example, been used to assess the lot-to-lot variability of raw medium powder and hydrolysates [31] or to characterise microbial or cell culture cultivation processes in different reactor scales [32,33,34,35,36,37]. Besides PCA, a widely used regression-based MVDA approach is the partial least-squares (PLS) regression [38]. During the calibration of PLS regression models, latent variables (LVs) are generated, which iteratively maximise the covariance between the independent, online data and dependent, offline data. The resulting linear relationship can then be used to calculate the respective offline parameter value from unknown spectral data. The implementations of PLS regression used for the online monitoring and control of bioprocesses in stirred tank reactors are numerous and have extensively been reviewed in the literature [26,39,40]. Yet, to date, the means for the online recording of high-dimensional data in microbioreactors are limited, and applications of PLS modelling are mostly based on spectral at-line measurements [19,20,41,42,43]. Recently, Sawatzki et al. [44] used PLS regression to predict the endopolygalacturonase activity during fed-batch cultivations of *Saccharomyces cerevisiae* in 8–12 mL stirred cultures. In the study, the PLS model relied on a combination of online and automated at-line measurements. One microbioreactor system, which can provide high-dimensional online data suitable for PLS modelling, is the MTP-based BioLector^®^ system. However, the currently commercially available system is limited in the number of filters used for the measurement of fluorescence and scattered light measurements. In a more recent advancement, Ladner et al. [45] implemented a monochromator-based excitation wavelength scan in combination with a charge-coupled device (CCD) camera for the measurement of highly-resolved emission spectra. The setup allows for the multi-wavelength (2D) fluorescence monitoring of each well of an MTP. In the initial study demonstrating the new setup, the generated 2D spectra were used to generate PLS regression models for calculating characteristic process parameters during batch cultivations of *Hansenula polymorpha* and *Escherichia coli* [45]. In a subsequent study, PLS regression was combined with a mechanistic modelling approach to completely avoid offline measurements for PLS model calibration [46]. In a third study, the setup was used to conduct multi-wavelength online monitoring of the scattered light during co-cultures of *Lactococcus lactis* and *Kluyveromyces marxianus* [47]. Here, PLS modelling was shown to be applicable for the determination of the biomasses of the two individual organisms within the co-culture.

This paper extensively elaborates on the application and limits of 2D fluorescence online monitoring in MTPs using the MVDA methodologies of PCA and PLS. A comprehensive experimental dataset of *H. polymorpha* cultivations is generated, consisting of online 2D fluorescence spectra and offline measurements of the key process parameters of glycerol, cell dry weight (CDW), and pH value. The experiment is conducted at an elevated throughput with a total of eight different initial cultivation conditions comprising varying glycerol concentrations and CDWs. Based on the online and offline measurements from two replicates of two different cultivation conditions, individual PLS regression models for the three key process parameters are generated. PLS model validation is conducted using additional offline samples for the two different cultivation conditions, which were not used for the model generation. Subsequently, model transferability is demonstrated by applying the generated PLS models to spectra of unknown cultivation conditions. Based on the modelling results, the applicability of the validation approach, as well as the necessity for spectral replicates, are discussed, finally leading to a revised experimental layout with reduced sampling efforts. 

## 2. Materials and Methods

### 2.1. Microorganism 

*Hansenula polymorpha* RB11 pC10-*FMD* (P*_FMD_*-GFP), expressing green fluorescent protein (GFP), was maintained at −80 °C in modified SYN6-MES medium [48] supplemented with 150 g/L glycerol.

### 2.2. Media Composition

A modified SYN6-MES mineral medium based on Jeude et al. [48] was used for pre and main cultures. The basic solution consisted of 1.0 g/L KH_2_PO_4_, 7.66 g/L (NH_4_)_2_SO_4_, 27.3 g/L 2-morpholinoethanesulfonic acid (MES), 3.0 g/L MgSO_4_·7H_2_O, 3.3 g/L KCl and 0.3 g/L NaCl. Before sterilisation at 121 °C for 20 min, the pH was adjusted to 6.0 using 1 M NaOH. For supplementation of the basal solution, a concentrated, sterile-filtered trace-element solution was added to provide 0.65 mg/L NiSO_4_·6H_2_O, 0.65 mg/L CoCl_2_·6H_2_O, 0.65 mg/L H_2_BO_4_, 0.65 mg/L KI and 0.65 mg/L Na_2_MoO_4_·2H_2_O. A sterile microelement solution was supplemented to provide 66.5 mg/L EDTA (Titriplex III), 66.5 mg/L (NH_4_)_2_Fe(SO_4_)_2_·6H_2_O, 5.5 mg/L CuSO_4_·5H_2_O, 20 mg/L ZnSO_4_·7H_2_O and 26.5 mg/L MnSO_4_·H_2_O. A final concentration of 1.0 g/L CaCl_2_·2H_2_O was supplemented from a sterile stock solution. Additionally, a sterile vitamin solution was added to supply 0.4 mg/L d-biotin and 133.4 mg/L thiamine · HCl. For the preparation of the stock solution, the d-biotin was first dissolved in a 10 mL mixture (1:1) of 2-propanol and deionised water and then added to the thiamine hydrochloride, dissolved in 90 mL deionised water. Glycerol was added to the media from a sterile 500 g/L stock solution to achieve the respective final glycerol concentration. Sterile water was added to account for differences in volumes.

### 2.3. Precultures

Precultures were carried out in 250 mL shake flasks using a shaking frequency (n) of 350 rpm, a shaking diameter (d_0_) of 50 mm, and a filling volume (V_L_) of 10 mL. The medium was inoculated with an initial optical density measured at a wavelength of 600 nm (OD_600_) of 0.1 and cultivation was carried out at a temperature (T) of 30 °C. The initial glycerol concentration was 10 g/L. The precultures were harvested after growth had stopped, indicated by oxygen transfer rate (OTR) measurements. Thereby, carryover of glycerol from the preculture to the main culture was avoided. Additionally, before inoculation of the main culture media, the cells were washed in fresh, glycerol-free medium.

### 2.4. Main Cultures

**Figure 1:**
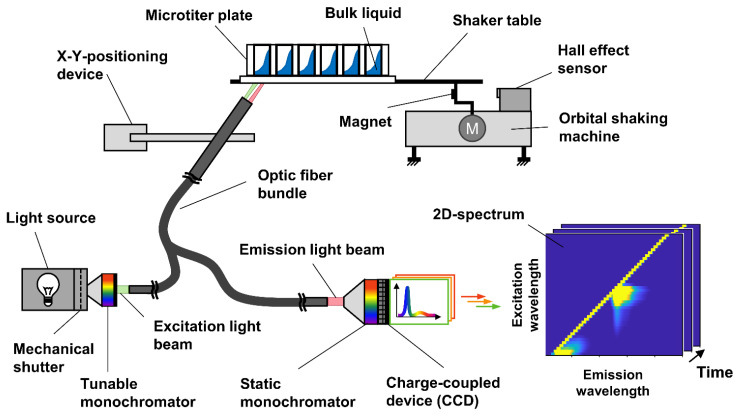
Schematic setup of cultivation system for 2D fluorescence online monitoring in microtiter plates. The shaker table provides continuous shaking of the microtiter plate (MTP). The optical fibre is displaced below each well of the MTP by an X–Y-positioning device. With the tuneable monochromator, the excitation wavelength is selected and sent to the MTP via the optical fibre. The emitted light is collected by the fibre and sent to a second static monochromator. The static monochromator disperses the emitted light into its constituent wavelengths. The resulting emission spectrum is recorded by a charge-coupled device (CCD) camera. To guarantee a reproducible shaker-table position during measurement, the spectral measurement of the CCD camera is triggered by a hall sensor. The emission spectra are assembled into 2D spectra for each excitation wavelength scan of each well over the cultivation time. Modified from [45] and used with permission from publisher.

**Figure 2:**
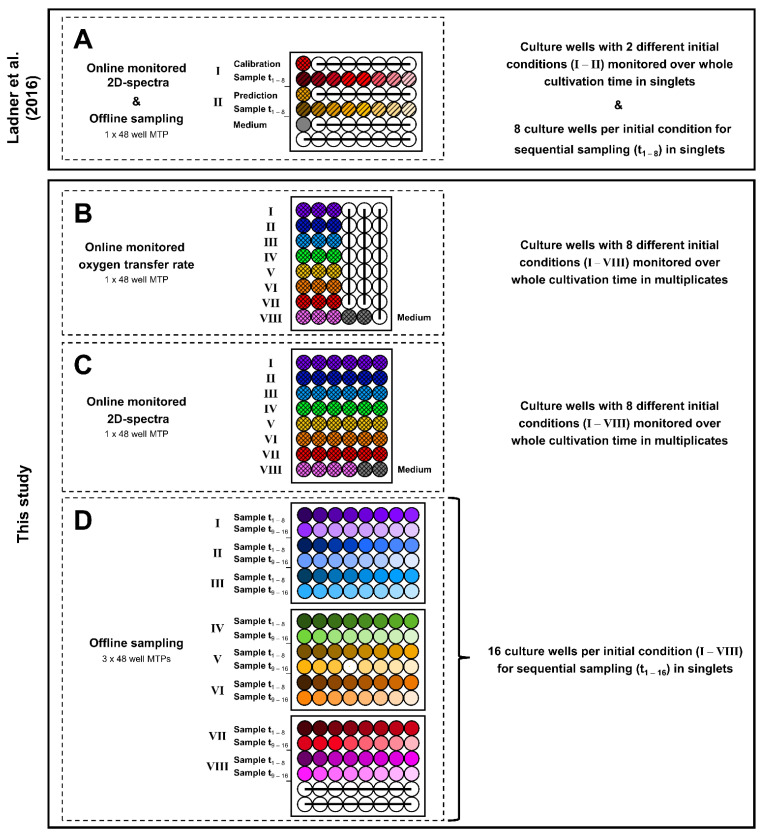
Comparison of experimental sampling strategies used in previous and current publication. The previous publication by Ladner et al. [45] included direct sampling from an MTP in the online-monitoring cultivation system, limiting both the amount of online data and offline samples (**A**). This study used multiple MTPs in parallel cultivation devices (**B**–**D**), indicated by dashed boxes. The metabolic activity in the form of the oxygen transfer rate (OTR) was measured in the µRAMOS cultivation system published by Flitsch et al. [49] (**B**). Recording of 2D spectra (**C**) and offline sampling (**D**) were conducted in two individual devices. Cross-hatched wells indicate cultures monitored over the whole cultivation time. Diagonal-hatched wells indicate monitored wells being sampled during cultivation. Unhatched wells indicate non-monitored wells used for sampling. Grey wells indicate wells with non-inoculated medium. White, struck-through wells were not used.

Main cultures were carried out in 48-round-well microtiter plates with a transparent bottom (MTP-R48-B, Beckman Coulter GmbH, Baesweiler, Germany). In contrast to the studies conducted in the 2D fluorescence cultivation system by Ladner et al. [45] (Figure 1 and Figure 2A), in this study, multiple cultivation systems including multiple MTPs were used (Figure 2B–D). To provide identical starting conditions for each cultivation condition, indicated by the colours in Figure 2, the medium was supplemented with the respective volume of glycerol and inoculum. The resulting “master mixes” were distributed over the MTPs. Afterwards, for the OTR-monitored cultures (Figure 2B), a polyolefin-based gas-permeable membrane (900371-T, HJ-Bioanalytik GmbH, Erkelenz, Germany) was used as a sterile barrier, according to Flitsch et al. [49]. The aeration rate of humidified air was set to 1.0 vvm. The MTPs for spectroscopically monitored (Figure 2C) and sampled cultures (Figure 2D) were sealed with a gas-permeable membrane (AeraSeal film, A9224-50EA, Sigma-Aldrich Chemie GmbH, Steinheim, Germany) and incubated in a humidified atmosphere. For all MTPs, the filling volume (V_L_) of each well was 0.8 mL. During cultivation, all MTPs were incubated at 30 °C, and continuously shaken at a shaking frequency of 1000 rpm and a shaking diameter of 3 mm. 

### 2.5. Online Monitoring

The spectral online monitoring was conducted using the MTP cultivation system introduced by Ladner et al. [45] as shown in Figure 1.

A Y-shaped optical fibre bundle was sequentially displaced below each well of a continuously shaken 48-well microtiter plate using an X–Y-positioning device. At the measurement position, 2D spectra were acquired by cycling through discrete excitation wavelengths using an automated grating-based monochromator (CP140-1602, Horiba Jobin Yvon GmbH, Bensheim, Germany) connected to the optical fibre. A spectral range from 280 nm to 700 nm with an increment step size of 10 nm was scanned. The light emitted from the culture within the well was collected by the optical fibre and transmitted to a second, static monochromator (microHR, Horiba Jobin Yvon GmbH, Bensheim, Germany), allowing for light dispersion and the subsequent recording of high-resolution emission spectra. For the spectral measurement using a CCD camera (Synapse, Horiba Jobin Yvon GmbH, Bensheim, Germany), an integration time of 30 ms was chosen. Reproducible measurements were ensured by a Hall effect sensor providing the information on the shaker-table position. After measurement, 2D spectra were generated by stacking the single-emission spectra according to the excitation wavelength, the respective well and measurement time. An intermediate 10 min break of measurements was included after measuring all 48 individual wells in one measurement cycle to reduce device wearing. As a result, one full 2D spectrum for each well of the 48-well MTP was obtained every 30 min.

The OTRs were measured using the Respiration Activity MOnitoring System (RAMOS) adapted for MTPs (µRAMOS). Oxygen-sensitive fluorescence spots are used in every individual well to determine the oxygen partial pressure (pO_2_) of the gas phase, using the Stern–Volmer equation for quenching. The OTR was determined as described by Flitsch et al. [49].

### 2.6. Determination of Offline Parameters

Cultures were regularly sampled from MTPs (MTP-R48-OFF, Beckman Coulter, Baesweiler, Germany) and shaken in a separate humidified incubator (ISF1-X, Adolf Kühner AG, Birsfelden, Switzerland) (Figure 2D). Sampling was conducted by harvesting the culture volume of one well at each sampling point. The complete culture volume was harvested without interrupting the shaking. At the starting time (t = 0 h), samples were taken from the main culture master mixes. The OD_600_ of the samples was determined in micro cuvettes (PS, Carl Roth, Karlsruhe, Germany) using a photometer (GENESYS 20, Thermo Scientific, Dreieich, Germany). Before measurement, the samples were diluted with a 0.9% (*w*/*v*) NaCl solution to allow a measurement between 0.1 and 0.3. The CDW was calculated from the OD_600_ using a linear correlation (Figure S1). The pH value was determined using a two-point calibrated pH meter (HI21, HANNA instruments Inc., Woonsocket, US, electrode: InLab Solids, Mettler Toledo GmbH, Columbus, OH, USA) and was measured in undiluted, non-centrifuged samples. For the determination of residual glycerol, the sample was centrifuged at 13,000 rpm for 5 min (SIGMA 1-15; Sigma Laborzentrifugen GmbH, Osterode am Harz, Germany). The supernatant was filtered using a 0.2 μm syringe filter (Acrodisc^®^ syringe filter 13 mm Minispike 0.2 μm GHP membrane, PALL GmbH, Dreieich, DE). Until the HPLC measurement was performed, samples were stored at 4 °C. The HPLC measurement (UltiMate3000, Dionex, Sunnyvale, CA, USA) was conducted using an organic acid resin column (250 × 8 mm; CS-Chromatographie Service, Langerwehe, Germany) at a flow rate of 0.8 mL/min and a temperature of 75 °C with 5 mM sulphuric acid. The final glycerol concentration was determined using a refractive-index detector (Shodex RI-101; Showa Denko Europe, Wiesbaden, Germany).

### 2.7. Spectral Data Processing 

The data preprocessing and multivariate data analysis were performed using Matlab 9.8.0.1380330 (R2020a) Update 2. For the initial spectral data reduction, the detected spectral range was reduced by only including emission wavelengths between -10 nm and +270 nm relative to the excitation wavelength. A moving average filter with a window size of 25 values was applied for every emission spectrum to reduce signal noise. The 2D spectra were converted to row vectors and reduced to every fifth wavelength combination. This accounts for a spectral resolution of 2 nm and is expected to represent the spectral information appropriately. Finally, each wavelength combination intensity (I) was referenced to the wavelength combination intensity of the first measurement cycle (I_0_) of the respective well by subtraction (I-I_0_).

### 2.8. Multivariate Data Analysis

For dataset handling, PCA, and PLS regression, the open-source Matlab toolbox *mdatools* [50] was used. For the temporal alignment of the spectral data from the online-monitored MTP (Figure 2C) and the offline measurements from the sampling MTPs (Figure 2D), the offline parameters for glycerol concentration, CDW and pH value were linearly interpolated between two sampling points. Subsequently, the interpolated offline parameter value, calculated for the time of spectral measurement, was extracted. 

For each offline parameter, PLS models were finally generated using the SIMPLS algorithm [51]. PLS model calibration was conducted using the interpolated data of the samples taken every 6 h. For the statistical evaluation of the models, the root-mean-square error (RMSE) of the respective dataset was determined. The RMSE of the calibration dataset was calculated twice. Firstly, the linear interpolation of the sample taken every 6 h of cultivation was used (RMSE_cal,6-h_). Secondly, the linear interpolation of the samples taken every 1.5 h of cultivation was used as the reference values (RMSE_cal,1.5-h_). The RMSE of the prediction dataset was calculated for the 1.5 h sampling interval (RMSE_pred,1.5-h_).

For the PLS model for glycerol, before PLS regression model generation, the residual glycerol concentrations were converted to total consumed glycerol concentrations, as this was proven to improve the predictability (data not shown). After applying the generated PLS model to the spectral data, the residual glycerol concentration was then recalculated from the initial glycerol concentration.

## 3. Results

### 3.1. Experimental Layout and Sampling Strategy

The hitherto studies, introducing 2D spectroscopy in MTPs, used a single MTP for the monitoring and sampling of only two or three different cultivation conditions per experiment [45,46] (Figure 2A). This significantly limited the experimental throughput, which otherwise is one of the huge advantages of the microbioreactor cultivation system. Further, the reduced throughput did not allow an extensive elaboration of the possibilities and limitations of 2D fluorescence monitoring in MTPs, which is an essential requirement for establishing a new online-monitoring technology. Hence, to increase the size of the generated datasets of *H. polymorpha* cultures to a total of eight different initial conditions, an experimental layout including a total of five 48-well MTPs in three parallel cultivation devices was chosen in this study (Figure 2B–D). The metabolic activity represented by the OTR was monitored online using the µRAMOS technology for MTPs [49] (Figure 2B). Further, the spectroscopic setup shown in Figure 1 was used to generate 2D spectra of each culture of a 48-well MTP every 30 min (Figure 2C). With up to six replicates per condition, the chosen layout also allows the evaluation of the reproducibility of the spectral measurements. For extensive sampling and offline measurement, three additional MTPs were incubated in a separate incubator (Figure 2D). With a total of 120 samples and up to 16 samples per cultivation condition, the progression for the concentration of glycerol, CDW, and pH value can be described in detail over the cultivation time. For the eight different cultivation conditions, the initial CDW and glycerol concentrations were varied. For the three initial cultivation conditions, a glycerol concentration of 9 g/L was chosen and the initial CDW was varied between 0.03 g/L to 0.13 g/L (Figure 2B–D, conditions I–III). For the remaining five initial cultivation conditions, the initial CDW was kept constant at 0.03 g/L, while the glycerol concentration was successively reduced to 0 g/L (Figure 2B–D, conditions IV–VIII).

### 3.2. Online and Offline Monitoring of Microtiter Plate Cultivations

**Figure 3:**
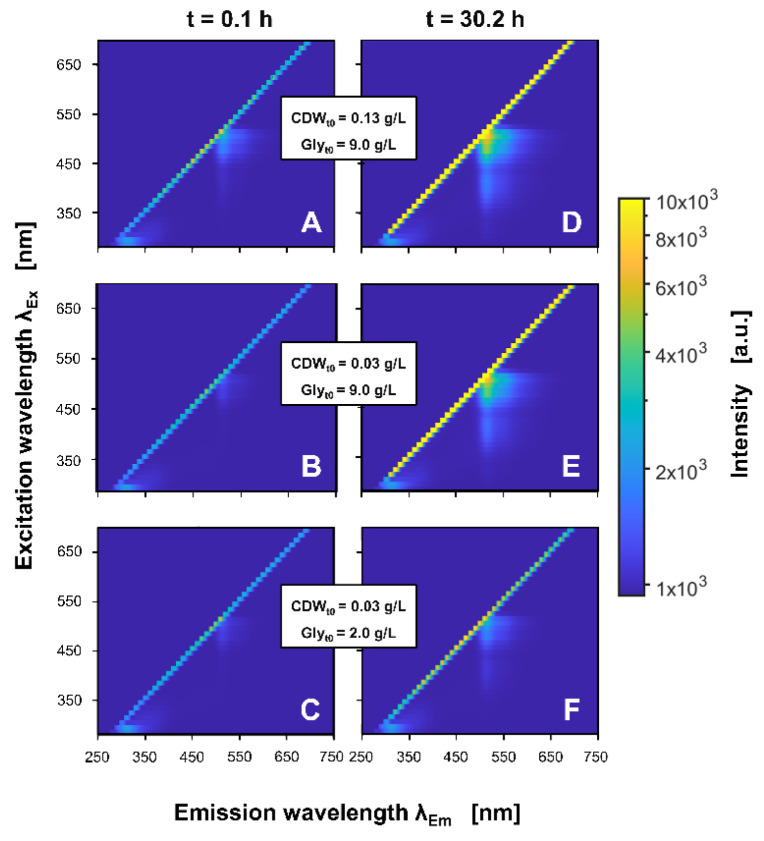
Exemplary 2D spectra of *H. polymorpha* RB11 pC9-*FMD* (P*_FMD_*-GFP) cultures in SYN6 medium after 0.1 h (**A**–**C**) and 30.2 h (**D**–**F**) for different initial cell dry weights (CDW_t0_) and glycerol concentrations (Gly_t0_). The spectra are recorded for cultures with 0.13 g/L CDW_t0_ and 9.0 g/L Gly_t0_ (**A**,**D**), 0.03 g/L CDW_t0_ and 9.0 g/L Gly_t0_ (**B**,**E**), and 0.13 g/L CDW_t0_ and 2.0 g/L Gly_t0_ (**C**,**F**). The spectra are smoothed according to the methods section, but not referenced to the first cycle. Spectroscopic measurement settings: excitation wavelength range = 280 nm–700 nm (step size = 10 nm), emission wavelength range = 278 nm–720 nm (step size = 0.45 nm), integration time = 30 ms. Cultivation conditions: 48-round-well microtiter plate, liquid volume = 800 µL, shaking diameter = 3 mm, shaking frequency = 1000 rpm, temperature = 30 °C.

In Figure 3, exemplary 2D spectra from individual wells of three different cultivation conditions after 0.1 h (Figure 3A–C) and 30.2 h (Figure 3D–F) of cultivation are shown (other conditions see Figure S2). 

The scattered light is represented by the bisecting line, describing identical wavelengths for excitation and emission. Fluorescence was observed in areas where the emission wavelength is higher than the respective excitation wavelength and, thus, to the right of the scattered light. The shift to a wavelength of lower energy, known as the Stokes shift, occurs as the absorbed energy is partially dissipated by a non-radiative processes [52]. The most prominent area of fluorescence was recognised for excitation wavelengths between 440 nm and 550 nm and emission wavelengths between 470 nm and 600 nm. This area can be ascribed to the fluorescence of GFP, which is expressed by the applied recombinant *H. polymorpha* strain [53,54]. The second area of fluorescence was observed at lower excitation wavelengths between 360 nm and 440 nm and comparable emission wavelengths between 470 nm and 550 nm. While this area is mainly connected to the fluorescence of intracellular flavines [55], spectral overlap from GFP fluorescence is also conceivable [53,56]. Comparing the spectra of the different cultivation conditions at the beginning of the cultivation, the GFP fluorescence intensity increased with an increasing initial CDW (Figure 3A–C). In contrast to that, after 30.2 h of cultivation (Figure 3D–F), the GFP intensities were observed to increase with the amount of the initially supplemented glycerol. For the cultures with high initial glycerol concentrations, after 30.2 h an additional area of fluorescence was observed at excitation wavelengths between 300 nm and 330 nm and emission wavelengths between 470 nm and 550 nm. Again, a relation to the flavines or the expressed GFP is conceivable [55,56]. Finally, increased fluorescence intensities were measured for excitation wavelengths between 280 nm to 350 nm and emission wavelengths between 290 nm and 360 nm. Although this area is commonly ascribed to the fluorescence of aromatic amino acids [57], the high intensities have previously been connected to the fluorescing polystyrene bottom of the microtiter plates [45,58]. This is partially supported by the 2D spectra of the non-inoculated medium shown in Figure S2F,L. In both 2D spectra, comparable fluorescence spots were detected in the respective spectral area, thus indicating a non-biogenic compound of constant concentration.

**Figure 4:**
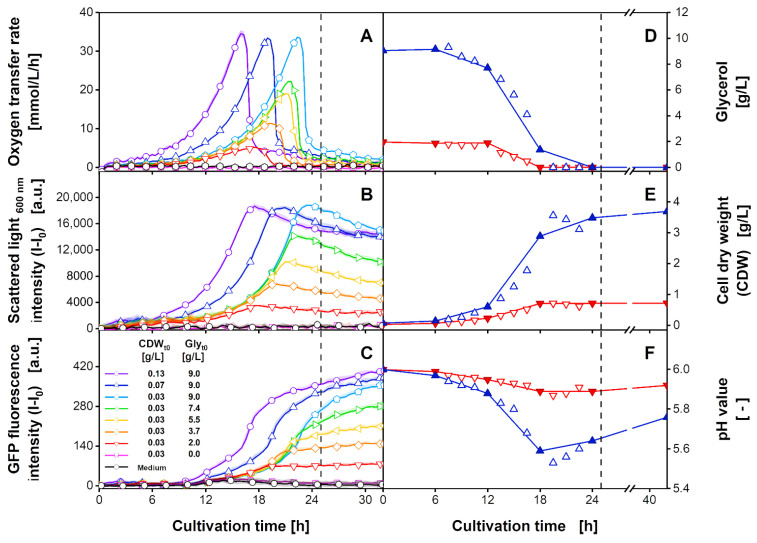
Time-resolved (**A**–**C**) online-monitoring signals and (**D**–**F**) offline sample measurements of *H. polymorpha* RB11 pC9-*FMD* (P*_FMD_*-GFP) cultivations in SYN6 medium at eight different initial cell dry weights (CDW_t0_) and glycerol concentrations (Gly_t0_). (**A**) Mean oxygen transfer rate (OTR) of cultures in triplicates determined by µRAMOS device [49]. Only every 5th data point is indicated by a hollow symbol for clarity. The standard deviation of triplicates is shown as shaded areas, which are barely visible due to good reproducibility. Singular online signals are shown in Figure S3. (**B**) Mean scattered light intensity at an excitation and emission wavelength of 600 nm and (**C**) mean GFP fluorescence intensity at an excitation wavelength of 420 nm and emission wavelength of 530 nm extracted from 2D spectra. Only every 5th data point is indicated by a hollow symbol for clarity. For inoculated cultures with glycerol concentrations above 0 g/L, scattered light and GFP signals describe mean values and standard deviation of six replicates, as described in Figure 2. For 0 g/L scattered light and GFP signals describe quadruplicates, while for medium wells the signals describe duplicates. Values of (**D**) glycerol, (**E**) cell dry weight (CDW), and (**F**) pH value for initial biomass and glycerol concentrations of 0.07 g/L and 9.0 g/L (blue) as well as of 0.03 g/L and 2.0 g/L (red) are based on singular HPLC and at-line measurement from parallel cultivation as described in Figure 2. Hollow symbols show offline measurements for 1.5 h sampling interval, whereas linearly interpolated, filled symbols indicate a 6 h sampling interval. Dashed vertical lines after 25 h indicate the last measurement included for PLS model generation, as explained in the text. Spectroscopic measurement settings: excitation wavelength range = 280 nm–700 nm (step size = 10 nm), emission wavelength range = 278 nm–720 nm (step size = 0.45 nm), integration time = 30 ms. Cultivation conditions: 48-well microtiter plate with round geometry, liquid volume = 800 µL, shaking diameter = 3 mm, shaking frequency = 1000 rpm, temperature = 30 °C.

For each of the recorded 2D spectra, the intensities of a singular wavelength combination can be extracted to describe the progression of the different cultures. In Figure 4, online signals of the OTR (Figure 4A), the scattered light at 600 nm (Figure 4B), and the GFP fluorescence intensity at an excitation wavelength of 420 nm and an emission wavelength of 530 nm (Figure 4C) are visualised over the cultivation time. The signals are averaged from three individual wells for the OTR measurement, and six individual wells for the spectral data. The shaded area describes the standard deviation of the replicates. The individual signals for each well are shown in Figures S3 and S4, respectively. 

The initially increasing OTR in Figure 4A indicates non-limited exponential growth. For the cultures with the highest glycerol concentration (Figure 4A, purple circles, blue upwards triangles, light blue pentagons), a maximum OTR of 33 mmol/L/h was measured, before sharply dropping to a value below 5 mmol/L/h. With decreasing glycerol concentrations, a flattened OTR curve and a reduced maximum value were observed. With increasing initial CDW (Figure 4A, light blue pentagons, blue upwards triangles, purple circles), the OTR increased earlier. Comparable to the OTR, for the culture with the highest glycerol concentrations, the scattered light signal at 600 nm increased exponentially up to maximum signal intensities of 18,000 a.u. (Figure 4B, light blue pentagons, blue upwards triangles, purple circles). Cultures with lower initial glycerol concentrations showed lower maximum scattered light values. When the peak in the OTR was reached, a slight asymptotical decrease in the scattered light signal was observed, which was more profound for higher initial glycerol concentrations. This observation is in good agreement with studies by Kottmeier et al. [59] and Kunze et al. [60]. These studies reported morphological changes of *H. polymorpha* and *K. lactis* under carbon-starvation conditions as a reason for the change in the scattered light signal. The GFP fluorescence signals at an excitation wavelength of 420 nm and an emission wavelength of 530 nm are shown in Figure 4C. An initial linear increase was observed after 7 h (purple circles), 9 h (blue upwards triangles), or 11 h (light blue pentagons, green rightwards triangles, yellow leftwards triangles, orange diamonds, pink squares) of cultivation, depending on the initial CDW. When the OTR began to decline and the scattered light entered a plateau, a sudden incline in GFP fluorescence intensity was observed for cultures with initial glycerol concentrations above 2 g/L. This sudden increase can be attributed to the de-repression of the formate dehydrogenase (*FMD*) promoter at low residual glycerol concentrations [61,62,63]. The de-repression results in an increased formation of GFP until the carbon source is fully depleted. Upon declining metabolic activity, the GFP fluorescence signal showed a transition to a linearly increasing signal, indicating residual GFP production. Alternatively, a change in the optical conditions within the broth is also conceivable.

**Figure 5:**
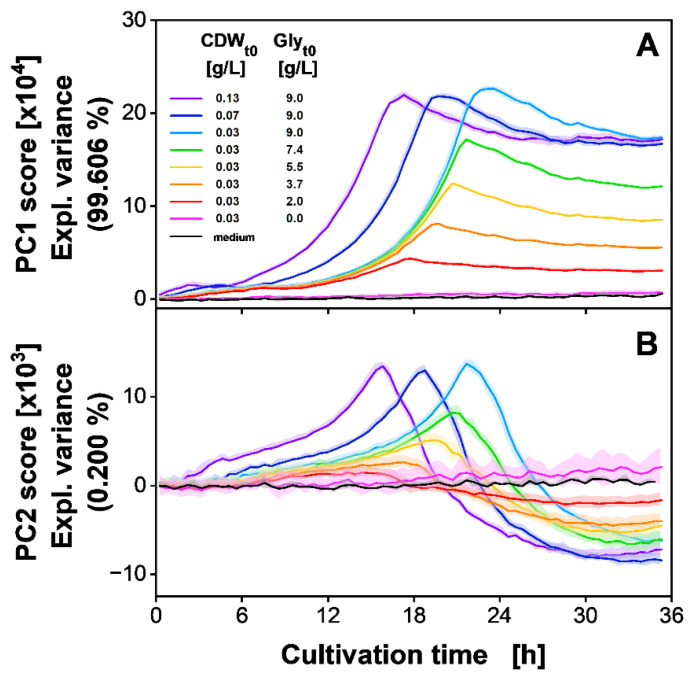
Scores of (**A**) first (PC1) and (**B**) second principal components (PC2) based on 2D spectra of *Hansenula polymorpha* RB11 pC10-*FMD* (P*_FMD_*-GFP) cultivations in SYN6 medium at eight different initial cell dry weights (CDW_t0_) and glycerol (Gly_t0_) concentrations. Signals show mean scores of six replicates. Standard deviation is shown as light shading. The individual scores are shown in Figure S4C,D. Spectroscopic measurement settings: excitation wavelength range = 280 nm–700 nm (step size = 10 nm), emission wavelength range = 278 nm–720 nm (step size = 0.45 nm), integration time = 30 ms. Cultivation conditions: 48-well microtiter plate with round geometry, liquid volume = 800 µL, shaking diameter = 3 mm, shaking frequency = 1000 rpm, temperature = 30 °C.

In Figure 4D–F, the residual glycerol concentration, CDW, and pH value are shown for the cultures with an initial CDW of 0.07 g/L and an initial glycerol concentration of 9 g/L (blue upward triangle) as well as for cultures with an initial CDW of 0.03 g/L and an initial glycerol concentration of 2.0 g/L (red downward triangle). As indicated, samples were taken every 1.5 h after the first 6 h (hollow symbols). The offline measurements follow the measured metabolic activity in Figure 4A, describing an exponential decrease for glycerol and an exponential increase for CDW. The pH value dropped during cultivation and rose as soon as glycerol was depleted. The offline data of a more practical 6 h sampling interval (filled symbols) were linearly interpolated and are indicated by a solid line. For the cultures with a lower initial glycerol concentration (Figure 4D–F, red line) the progression of the at-line parameters is reflected with only a slight deviation from the values of the 1.5 h sampling interval. However, for the culture with a higher initial glycerol concentration (Figure 4D–F, blue line), the linear interpolation of the 6 h sampling interval deviates from the progression described by the 1.5 h sampling interval. Inaccuracies are evident between 12 h and 24 h of cultivation. Here, the exponential course of the glycerol and CDW during the time of metabolic activity is poorly described. The depletion of the glycerol concentration is identified after 24 h instead of 19.5 h of cultivation. For the pH value, the linear interpolation of the 6 h sampling interval does not reproduce the minimal value of 5.53 measured after 19.5 h. Consequently, the rise in the pH value after glycerol depletion is also incompletely described. As demonstrated, the progression of the cultures can be interpreted by extracting the intensities as a single wavelength combination. However, this presupposes knowledge about the exact spectral area to be looked at. Further, it is possible that spectral information, which may be included in other parts of the 2D spectra, are not considered during this evaluation. Hence, in addition to evaluating individual spectral signals, principal component analysis (PCA) was applied for fast data exploration of the whole spectra. In Figure 5 the mean scores of the six replicates are plotted over the cultivation time. The individual scores are shown in Figure S4C,D.

As described before, the method is commonly used for reducing high-dimensional data to data with only a few principal components (PCs). The PCs are iteratively generated based on the maximum variance within the residing data. However, it is important to notice that data variance that has been described by a previous PC is no longer considered for a subsequent PC [29]. In practice, this approach helps to expose signal dynamics, which otherwise may be entangled with or covered by a more dominant signal. The scores of the first principal component (PC1) plotted over the cultivation time are shown in Figure 5A. The PC1 score accounts for 99.6% of the spectral information and is qualitatively identical to the scattered light signal at 600 nm (Figure 4B). The scores of PC2 in Figure 5B describe only 0.2% of the spectral variance. For the cultures with a high initial glycerol concentration (Figure 5B, purple, blue, light blue, green lines), the scores describe a linear increase during the first period of cultivation. Starting between 12 h and 17 h of cultivation, the scores increase non-linearly until metabolic activity ends, before finally dropping below the initial value. For the cultures with low initial glycerol concentration (Figure 5B, red, orange, yellow lines), the scores do not show the described change in the slope, but a transition into a plateau. Noticeably, for all individual cultures, the observed maximum PC2 score coincides with the sudden increase in the GFP fluorescence. This increase has previously been linked to the de-repression of the *FMD* promoter [62] and is more profound for the cultures of higher initial glycerol concentrations. The scores of PC1 and PC2 can be plotted against each other to allow the comparison of the cultivations without referring to the cultivation time. In Figure S5, the so-called score plot shows identical trajectories for the cultures of different initial CDW but identical glycerol concentrations (Figure S5, purple, dark blue, light blue lines). This indicates an identical growth pattern, which can also be observed in Figure 4A–C. For the cultures of lower initial glycerol concentrations, a comparable shape of trajectories is described. In general, the score plot confirms very good reproducibility of the singular replicates for all cultures.

### 3.3. PLS Regression Modelling

With the available online and interpolated offline data for the residual glycerol concentration, CDW, and pH value, PLS regression models can be generated. The resulting correlations can be used to calculate the respective offline values for the 2D spectra measured at non-sampled time points. This allows the shortening of the interval of available offline measurements from 6 h for sampling to only 30 min for the spectral measurement. Further, the model can be transferred to spectral data of cultivations, for which only the initial cultivation conditions (i.e., initial CDW and glycerol concentration) are known. In conclusion, this approach allows the description of the cultivation in more detail, while also reducing the necessary sampling efforts.

**Figure 6:**
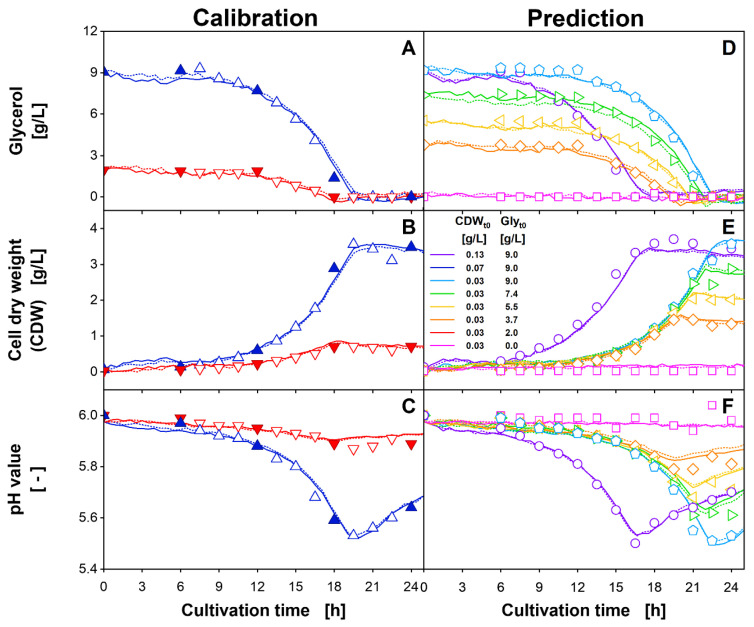
(**A**–**C**) Calibration and (**D**–**F**) prediction of the PLS model, generated by including two latent variables and linear interpolation for a 6 h sampling interval for (**A**,**D**) glycerol, (**B**,**E**) CDW and (**C**,**F**) pH value based on 2D spectra of duplicates. For calibration, a total of 212 2D spectra from duplicates of *H. polymorpha* RB11 pC9-*FMD* (P*_FMD_*-GFP) cultivations for initial biomass (CDW_t0_) and glycerol concentration (Gly_t0_) of 0.07 g/L and 9.0 g/L (blue) as well as of 0.03 g/L and 2.0 g/L (red) were included. The offline values used for linear interpolation and subsequent PLS model calibration are shown as filled symbols. The hollow symbols were used for model validation only. Solid and dotted lines describe the calculated parameter progression of duplicates. Cultivation conditions: 48-well microtiter plate with round geometry, liquid volume = 800 µL, shaking diameter = 3 mm, shaking frequency = 1000 rpm, temperature = 30 °C.

As can be seen from Figure 4A–C, spectral data were recorded for 32 h, although, for all cultures, the stationary phase was reached within 25 h. Thus, to avoid the recorded spectra of metabolically non-active cultures having a disproportional influence on the generated PLS model, only the first 25 h of cultivation were included in the datasets (see dashed vertical line in Figure 4). In total, the calibration datasets for generating the PLS models comprised 212 2D spectra, recorded for duplicates of two cultivation conditions. Arbitrarily, without further systematics, the culture with 0.03 g/L initial CDW and 2.0 g/L initial glycerol (Figure 4, red downward triangles), as well as the culture with 0.07 g/L initial CDW and 9.0 g/L initial glycerol (Figure 4, blue upward triangles) were chosen as the PLS calibration dataset. These two conditions represent the highest and second lowest initial glycerol concentrations tested, as well as two different initial CDWs. For the PLS model generation, further, the offline values extracted from the linear interpolation of the 6 h sampling interval (Figure 4D–F, filled symbols) were used. In addition to the calibration dataset, a prediction dataset was also generated to evaluate PLS model transferability. The prediction dataset consisted of the spectral data from cultures with initial conditions (CDW and glycerol concentration) that were not included in the calibration dataset (Figure 4A–C, purple circles, light blue pentagons, green rightward triangles, yellow leftward triangles, orange diamonds, pink squares). For each cultivation condition, two replicates were added to the respective dataset. In Figure S4A,B, the individual cultures included in the calibration and prediction dataset are highlighted in a dark colour. Following the high spectral collinearity, which was indicated by the high cumulated explained variance for PC1 and PC2 during the PCA (Figure 5), for all the offline parameters, PLS modelling was conducted using only two latent variables (LVs). For the statistical evaluation of the PLS models, the RMSE was calculated. For the calibration dataset, the RMSE was calculated based on the interpolation of the 6 h sampling interval (RMSE_cal,6-h_). Additionally, for model validation, the RMSE of the calibration dataset was also calculated using the linear interpolated values of the 1.5 h sampling interval (RMSE_cal,1.5-h_) as a reference. For the prediction dataset, the RMSE was calculated for the 1.5 h sampling interval (RMSE_pred,1.5-h_) to verify the successful transfer of the PLS model. An overview of the resulting RMSEs is given in Table S1A and Figure S6. The results of the final PLS models plotted over the cultivation time are shown in Figure 6. The course of the glycerol concentration calculated from the PLS model for the calibration and prediction datasets is described in Figure 6A,D, respectively. The course of the calculated CDW for the two datasets is shown in Figure 6B,E, while the pH value is described in Figure 6C,F. Comparable to Figure 4, the filled symbols indicate values of the practical 6 h sampling interval, which was used for the PLS model generation. The 1.5 h sampling interval, shown as hollow symbols, was only used for the calculation of the RMSE_cal,1.5-h_, and RMSE_pred,1.5-h_ and for validation.

The results for the PLS model of glycerol on the calibration dataset (Figure 6A) are in very good agreement with the offline measurements. In total, an RMSE_cal,6-h_ of 0.53 g/L, and an RMSE_cal,1.5-h_ of 0.31 g/L was obtained. This represents 5.9% and 3.5% of the glycerol concentration range (Table S1B). One reason for the reduced RMSE_cal,1.5-h_ becomes particularly obvious for the culture of 9 g/L glycerol (blue upward triangles). For this culture, the PLS model predicted glycerol depletion after 18 h, which aligns better with the values of the 1.5 h sampling interval than with the 6 h sampling interval. Therefore, with the PLS model being calibrated using the 6 h sampling interval, extensive overfitting of the interpolated offline data can be ruled out. Inaccuracies between the calculated glycerol concentration and offline sampling were observed during the first 6 h of cultivation. The biologically non-plausible fluctuations are comparable to the initial fluctuations of the PC1 and PC2 scores (Figure 5) and may have resulted from optical effects during low culture-broth turbidities [25]. In conclusion, they are an integral part of the spectral dynamics and were, therefore, also included in the generated PLS model using two LVs. 

The application of the PLS model for the glycerol concentration on the prediction dataset is shown in Figure 6D. For the cultures with the highest glycerol concentration (Figure 6D, purple circles, light blue pentagons), the concentrations were slightly underestimated between 6 and 10 h of cultivation. Further, inaccuracies were observed for the depletion of glycerol. Here, the final glycerol concentration of the culture with an initial CDW of 0.13 g/L and 9.0 g/L glycerol (Figure 6D, purple circles) was calculated to a value of 0.5 g/L. For cultures with lower initial glycerol concentrations, even negative final glycerol concentrations of −0.7 g/L were calculated (Figure 6D, green rightward triangles). In total, applying the PLS model to the prediction dataset resulted in an RMSE_pred,1.5-h_ of 0.34 g/L. With a relative value of 3.8% of the glycerol concentration range, this represents an increase in the RMSE of only 0.3% compared to the RMSE_cal,1.5-h_. This result supports very good model transferability (Table S1B).

In addition, for the PLS model of CDW, which is depicted in Figure 6B, good predictability and limited overfitting were indicated. The RMSE_cal,6-h_ of 0.19 g/L, and the RMSE_cal,1.5-h_ of 0.12 g/L describe a relative RMSE of 5.1% and 3.2% of the measured CDW range. This is well within the range of the expected error for gravimetric CDW determination [64,65]. The reduced value for the RMSE_cal,1.5-h_ can be explained by the very accurate alignment of the calculated values and the offline samples of the 1.5 h sampling interval for the cultures of higher glycerol concentration (Figure 6B, blue upward triangles). Slight deviations between the measured offline samples and the calculated values were again observed during the first 6 h of cultivation. Noticeably, at the end of the cultivation, the PLS model calculated a stagnant CDW, while the scattered light intensity at 600 nm (Figure 4B) decreased after glycerol depletion. This deviation resulted from the ability of the PLS regression algorithm to vary the importance for a single input variable, i.e., wavelength combination [66]. Thereby, the contribution of a spectral signal with strong deviation from the measured offline values, such as a non-stagnant signal progression after glycerol depletion, can be reduced. 

Applying the generated PLS regression model to the prediction dataset resulted in an RMSE_pred,1.5-h_ of 0.14 g/L, constituting 3.9% of the CDW range. One reason for the increased RMSE_pred,1.5-h_ can be found in the systematic underestimation of the CDW, which was especially observed for the cultures with the highest initial CDW concentration (Figure 6E, purple circles). The increase of 0.7% in comparison to the RMSE_cal,1.5-h_, again indicates good transferability (Table S1B).

In comparison to the other offline parameters, the pH value did not describe monotonous behaviour. Nevertheless, the results of the PLS model (Figure 6C) are in very good agreement with the determined trajectory of the offline measured values. Strikingly, even though the overall minimum pH value of 5.53, which was measured after 19.5 h of cultivation (Figure 6C, blue hollow upward triangle), was not part of the practical 6 h sampling calibration dataset, the generated PLS model predicted the respective pH value correctly. In total, the PLS model resulted in an RMSE_cal,6-h_ of 0.032, and an RMSE_cal,1.5-h_ of 0.023. Thus, with 6.0% and 4.3% of the offline parameter range, the relative RMSE_cal,1.5-h_ of the pH value was slightly higher than for the glycerol and CDW concentration (Table S1B). The highest deviations between the calculated and the offline measured pH values of the 1.5 h sampling interval were observed between 12 h and 18 h of cultivation for the culture with 9 g/L glycerol (Figure 6C, blue upward triangle). Additionally, for the culture with 2 g/L initial glycerol (Figure 6C, red downward triangle) a systematic offset was calculated for the pH value after 18 h of cultivation. Here, differences of up to 0.04 pH units were determined. The application of the PLS model to the prediction dataset (Figure 6F) resulted in an RMSE_pred,1.5-h_ of 0.029, accounting for 5.3% of the pH value range. Comparable to the culture with 2 g/L glycerol used for calibration (Figure 6C, red downward triangles), as well as for the prediction dataset, a systematic overestimation of the pH value was observed for the cultures with initial glycerol concentrations below 9 g/L (Figure 6F, green rightward triangles, yellow leftward triangles, orange diamonds) during the phase of increasing pH value. Nevertheless, considering the increase of 1% of the parameter range in comparison to the RMSE_cal,1.5-h_, good model transferability can be assessed (Table S1B).

**Figure 7:**
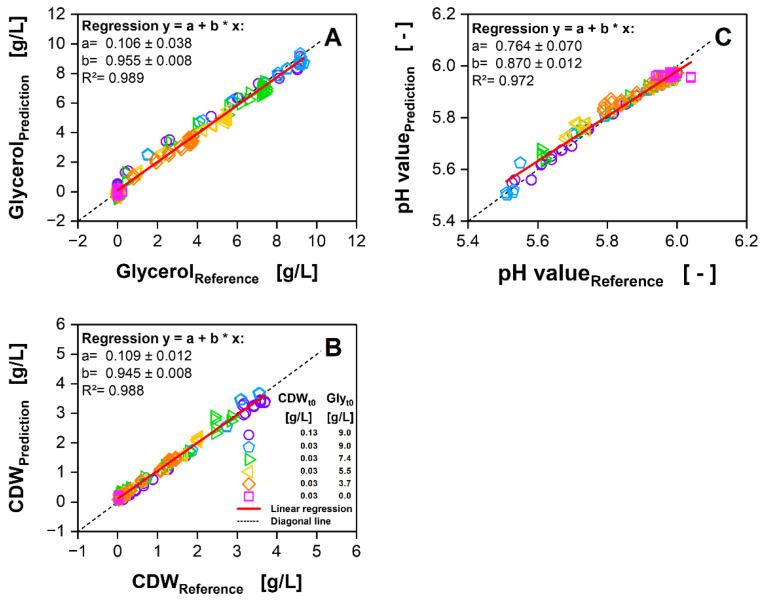
Parity plots for the duplicate-based PLS prediction of offline parameters (**A**) glycerol, (**B**) cell dry weight (CDW) and (**C**) pH value, using two latent variables on 2D spectra of duplicates of *H. polymorpha* RB11 pC9-*FMD* (P*_FMD_*-GFP) cultivations in SYN6 medium at six different initial cell dry weights (CDW_t0_) and glycerol concentrations (Gly_t0_). Only data points with corresponding offline sampling data, indicated by a hollow symbol in Figure 6, are plotted. Red line shows resulting linear correlation between predicted values and reference offline values.

For all the offline parameters, the parity plots in Figure 7 further summarise the PLS results for the prediction dataset. Data points are only shown for the spectral measurement time points, which are closest to the offline sampling time point of the 1.5 h interval (Figure 6D–F, hollow symbols).

For the determination of the reference offline value, the linear interpolation of the 1.5 h sampling interval was used. In parallel to Figure 6D, in Figure 7A, an overestimation is indicated for low glycerol concentrations, while for the time point of the depletion of glycerol, an underestimation is observed. For high concentrations representing the earlier part of the cultivation, the data are evenly scattered around the diagonal line. Overall, an R² value of 0.982 is calculated. For the prediction of CDW (Figure 7B), the R² is determined to be 0.988. Here, the slight underestimation at high CDWs and the overestimation at low CDWs can be identified as a small systematic error. The pH value (Figure 7C) is the only offline parameter showing an inflection over the cultivation time. This leads to a lower number of data points for lower pH values, while more data points for higher pH values are available. In consequence, this displaced distribution leads to the lowest R² of 0.972.

### 3.4. Impact of Numbers of Latent Variables

The ability to transfer the generated PLS models to an unknown prediction dataset is critical for high-throughput experiments in MTPs. One parameter with a high impact on the transferability is the number of latent variables (LVs). With increasing LVs, the independent (spectral) and dependent (offline) data of the calibration dataset are fitted more closely to one another as the amount of included covariance increases. However, PLS models with a high number of LVs are prone to overfitting, leading to the inclusion of non-representative signal variations, such as spectral noise [66,67,68]. The optimal number of LVs is usually identified using external validation datasets or internal cross-validation methods. For the latter, PLS models are repeatedly generated from a random or systematically chosen subset of the calibration dataset, before being applied to the remaining subset. The number of LVs is then chosen according to a minimum RMSE of the cross-validation subset [69,70,71]. In this study, the appropriate number of LVs was identified using the 1.5 h sampling interval for validation. In theory, a decrease in the RMSE_cal,6-h_ together with an increase in the RMSE_cal,1.5-h_ hints at overfitting of the calibration data, as the 1.5 h sampling interval is expected to describe the data more accurately.

The direct comparison of the RMSE_cal,6-h_ and the RMSE_cal,1.5-h_ for increasing LVs is shown in Figure S6. Additionally, the RMSE_pred,1.5-h_ is shown for external validation. As expected, for all the offline parameters, the RMSE_cal,6-h_ (Figure S6, green circles) decreased with increasing LVs. The RMSE_cal,1.5-h_ (Figure S6, black squares) and the RMSE_pred,1.5-h_ (Figure S6, red upward triangles) showed a non-monotonous pattern with a local minimum value. For the glycerol concentration and the CDW, the previously chosen number of two LVs represented the lowest values for both the RMSE_cal,1.5-h_ and the RMSE_pred,1.5-h_. For the pH value, the minimum RMSE_cal,1.5-h_ was observed for three LVs. For all the offline parameters, the RMSE_pred,1.5-h_ obtained for six LVs was higher than the respective minimum value.

The impact of overfitting due to an excessive number of LVs was exemplarily illustrated by the PLS models of the CDW. When increasing the number of LVs from two to six, the RMSE_cal,6-h_ decreased from 0.19 g/L to 0.12 g/L. This represents a reduction of 1.8% of the CDW range (Table S1B). In parallel, the RMSE_pred,1.5-h_ increased by 1.8% as the value increased from 0.14 g/L to 0.21 g/L. The resulting trajectories of the models are shown in Figure S7. As expected, the noise of the calculated offline values increased for the PLS model including six LVs (Figure S7B) in comparison to two LVs (Figure S7A). Additionally, the calculated trajectory of the calibration dataset showed a slight yet systematic overestimation between 12 and 18 h of cultivation. This overestimation led to a closer approximation of the interpolated 6 h sampling interval, which in turn decreased the RMSE_cal,6-h,_ but also increased the RMSE_cal,1.5-h_. Transferring the model of six LVs to the prediction dataset (Figure S6C,D) led to poor model performance. Especially, the calculated values of the culture with an increased initial CDW (Figure S7D, purple circles) strongly deviated from the offline measurement during the last phase of the cultivation. Additionally, the values for the replicates were less reproducible. This decrease in model performance underlines the necessity for an appropriate way to choose the number of LVs. Here, the approach of using two sampling intervals was shown to be effective, as it led to very comparable RMSE_cal,1.5-h_ and RMSE_pred,1.5-h_.

### 3.5. Impact of Averaging Spectral Data of Technical Duplicates

As presented above, successful PLS modelling was achieved using only two technical replicates. However, the available spectral data of six replicates can be used to evaluate the impact of signal noise on the PLS results. To do so, new spectral datasets for both the calibration and the prediction dataset are generated by averaging the 2D spectra of six replicates per measurement time. Subsequently, PLS models are again generated based on the 6 h sampling interval using two LVs (Figures S8 and S9). As expected, due to the averaging step, less noise is observed for the trajectories of the revised models. Additionally, for all the offline parameters, the RMSEs of the six-replicate-average-based model are improved between 2.7% and 0.1% of the respective offline parameter range, compared to the previous duplicate-based model (Table S1B). The evaluation of the number of LVs is shown in Figure S6D–F. For the CDW and the pH value, again, the minimum RMSE_cal,1.5-h,_ and RMSE_pred,1.5-h_ were calculated for two and three LVs, respectively. Whereas, for the glycerol concentration, the minimum was determined for four LVs. Comparable to the duplicate-based modelling, the optimal number of LVs can be identified according to the minimum of the RMSE_cal,1.5-h_ as well as by a strongly decreasing RMSE_cal,6-h_. 

Finally, the RMSE_pred,1.5-h_ resulting from the optimal number of LVs for the respective spectral dataset can be compared. For the glycerol concentration, the relative RMSE_pred,1.5-h_ decreased by 1.0% when using the six-replicate-average-based PLS model including four LVs instead of the duplicate-based PLS model including two LVs. For the PLS model of CDW, the RMSE_pred,1.5-h_ of the six-replicate-average-based dataset improved by 0.8%. For both spectral datasets, the optimum was observed for two LVs. Finally, for the pH value, an improvement of only 0.17% was achieved by using three LVs for the six-replicate-average-based model instead of six LVs for the duplicate-based model. In conclusion, these limited improvements do not outweigh the added value of an increased experimental throughput if only two parallel cultures are performed per cultivation condition.

## 4. Discussion

This study clearly presents that the PLS modelling performance for the *H. polymorpha* cultivations is dependent on the experimental strategy, dataset composition, and data processing. This dependency becomes even more striking with regard to the modelling results by Ladner et al. [45] and Paquet-Durand et al. [46]. Although the reported absolute RMSE of calibration and prediction for these studies are mostly of the same order (Table S2), the individual experimental approaches differ significantly.

For example, the study by Ladner et al. [45] used the same MTP for online monitoring and offline sampling. This reduced the experimental throughput to only two different initial cultivation conditions (Figure 2A). For the PLS modelling, the calibration dataset contained spectral data from a single culture replicate of the first initial cultivation condition. The prediction dataset consisted of spectral data of the second initial cultivation condition and was used for model validation. For PLS model generation, the number of LVs was chosen according to a minimised RMSE for the prediction dataset. Thereby, the PLS model solely relied on the covariance between the 2D spectra of a single culture and the determined offline values. This in turn increased the risk of including replicate-specific spectral fluctuations into the PLS models. As a result, model transferability is expected to be decreased, as indicated by a high difference between the RMSE of the calibration and the prediction dataset (Table S2, [45]). By varying the sampling intervals instead of splitting the calibration and validation datasets by batches, in the present study, model calibration and validation could be conducted with 2D spectra of two cultivation conditions. Thereby, identical states of cultivation (e.g., a specific pH value) were described by multiple cultures at different metabolic states (e.g., biomass concentration). In combination with the increased number of replicates, this redundancy prevented the final PLS model from including unrelated signal features, such as the declining scattered light signal observed after the depletion of glycerol. Consequently, this approach led to a better model transferability as indicated by the more comparable RMSE_cal,1.5-h,_ and RMSE_pred,1.5-h_.

In addition to the dataset composition, the interpolation of the offline values also differs between the studies. The linear interpolation used in this study represents the least sophisticated interpolation method and was chosen for simplicity reasons. Yet, especially for the 6 h sampling interval, which led to an acceptable effort in the laboratory, the exponential growth dynamics were described inaccurately. During PLS model calibration, these inaccuracies could be avoided by using a low number of LVs, which limited the extent of overfitting the interpolated values. While this resulted in a comparably high RMSE_cal,6-h_, very good model performance was indicated by a comparable RMSE_cal,1.5-h,_ and RMSE_pred,1.5-h_. In the study of Ladner et al. [45], splines were used for the interpolation of the offline values. This provided the ability to approximate non-linear signal dynamics without the need for additional process knowledge. For the 3 h sampling interval this method is not expected to significantly change the overall PLS model outcome, but rather locally reduces the residuals. For longer offline sampling intervals, however, this approach may hold a significant advantage over linear interpolation. Finally, Paquet-Durand et al. [46] used mechanistic modelling for calculating the trajectories of the offline values over the cultivation time. By using a priori process knowledge about the interconnection of biomass and substrate concentration, the overall growth dynamics were summarised in form of parameters such as the growth rate and the biomass yield. The parameters were then calculated based on the sampling values using a least-square fit. In contrast to the interpolation methods of the other studies, this fitting approach can overcome a certain degree of inaccuracy of the singular sample values. Therefore, it is expected to approximate microbial growth more accurately in comparison to the other interpolation approaches.

**Figure 8:**
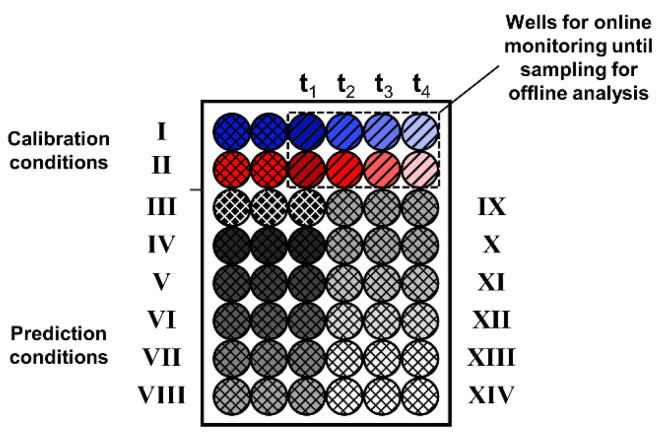
Visualisation of the revised experimental layout providing an increased throughput including two calibration (I–II) and twelve prediction conditions (III–XIV). Diagonal-hatched wells (t_1_–t_4_) indicate wells monitored online until time of sampling. Cross-hatched wells show wells being monitored online by 2D fluorescence over the whole cultivation time. Coloured wells describe cultivation conditions used for calibration. Grey- to white-shaded wells indicate conditions included in the prediction dataset.

Following these considerations and the results of the presented study, the initial experimental layout (Figure 2B–D) can be revised. Most importantly, the monitoring of six replicates, leading to a strong reduction in experimental throughput, becomes unnecessary. For the spectral averaging, only a limited improvement of the resulting RMSE (Figure S6D–F) and the calculated trajectories (Figure S8D–F) was found. Thus, for a revised experimental layout, a maximum of three replicates is expected to be sufficient. Additionally, only a fraction of offline samples needs to be taken, as the approach of using two sampling intervals was found to result in well-transferable PLS models. Consequently, the previous experimental setup can be reduced to a single MTP that is used for spectral online monitoring and offline sampling, as shown in Figure 8. 

In the revised experimental layout, for each initial cultivation condition of the calibration dataset, two wells are reserved for complete spectral monitoring (Figure 8, cross-hatched wells). Additionally, four wells are monitored until being sampled during the cultivation (Figure 8, diagonal-hatched wells). By taking an initial sample from the master mix and a final sample from one of the completely online-monitored wells, the total number of samples per calibration condition is increased to six. With these six samples, the previously implemented 6 h sampling interval can be reproduced. Comparable to this study for PLS model calibration, the offline values of the two cultivation conditions can be linearly interpolated. To provide an accurate description of the offline parameters for validation, spline interpolation or mechanistic modelling can alternatively be used. Following this approach, the experimental throughput of the prediction dataset can be increased to up to twelve different cultivation conditions in triplicates (Figure 8, conditions III–XIV). For external PLS model validation, samples of the culture conditions of the prediction dataset could be taken at the beginning and the end of the experiment. Furthermore, as triplicates are used for each cultivation condition, an additional validation sample could be taken throughout the cultivation. In conclusion, the extensive manual sampling effort, which was conducted in this study, can be averted, while the PLS model can still be validated. In detail, the revised experimental layout reduces the number of manual samples from 120 to 48. This represents a 2.5-fold reduction, while still providing sampling for external validation. In parallel, the experimental throughput is increased 1.75-fold from 8 to 14 cultivation conditions.

## 5. Conclusions

This study presents a thorough evaluation of the 2D fluorescence online monitoring of *H. polymorpha* batch cultures in microtiter plates. In addition to the online monitoring, an extensive sampling effort with a total of 120 single samples was conducted and subsequently evaluated using multivariate data analysis (MVDA) methods. For the recorded spectral data, PCA was used to expose the dynamics, which were otherwise covered by biomass-related effects. Further, by plotting the scores of PC1 against PC2, cultivations could be compared regardless of the cultivation time. PLS models for the offline parameters of glycerol, CDW, and pH value were generated for a practical 6 h sampling interval and subsequently validated based on a 1.5 h sampling interval. This led to an excellent transferability of the PLS models to the prediction datasets. The RMSE_pred,1.5-h_ of 0.34 g/L for glycerol, 0.14 g/L for CDW, and 0.029 for the pH value were very comparable to the RMSE_cal,1.5-h_. Furthermore, the variation of LVs was presented to describe the balance between over- and underfitting. A procedure for choosing a feasible number of LVs by using the two different sampling intervals for model validation was suggested. Finally, using average spectral data from six replicates was shown to only marginally improve the PLS model performance. Therefore, the initial experimental setup was revised, allowing the assessment of up to 14 different cultivation conditions within a single MTP experiment, while additionally reducing the number of offline samples.

In conclusion, this study represents a comprehensive benchmark dataset that can be used for comparison with future experiments using MVDA for the evaluation of online-monitored cultures in MTPs. Especially for cultivation conditions that are less related to one another, such as during media or strain screening experiments, the PLS model performance is expected to decrease. In addition, with the fluorescence and scattered light signals becoming more convoluted for more complex experimental setups, a systematic approach for determining the ideal conditions of the calibration dataset must be developed. Finally, for the confident minimisation of sampling efforts, the different model validation approaches must be evaluated. 

## Data Availability

The data that support the findings of this study are available from the corresponding author upon reasonable request.

## Supplementary Materials

The following supporting information can be downloaded at: https://www.mdpi.com/article/10.3390/bioengineering9090438/s1, Table S1: Absolute and relative RMSE resulting for the PLS model for the glycerol concentration, CDW and pH value based on batch cultures of *Hansenula polymorpha* RB11 pC10-*FMD* (P*_FMD_*-GFP) at different initial CDW and glycerol using a variable number of LVs.; Table S2: Overview of the absolute and relative RMSE reported for the PLS models generated for batch cultures of *Hansenula polymorpha* RB11 pC10-*FMD* (P*_FMD_*-GFP) at different initial CDW and glycerol concentrations. Figure S1: Linear correlation between cell dry weight (CDW) and optical density at 600 nm (OD600); Figure S2: Exemplary 2D spectra of *Hansenula polymorpha* RB11 pC10-*FMD* (P*_FMD_*-GFP) cultures in SYN6 medium after 0.1 h (A–E) and 30.2 h (G–J) for different initial cell dry weights (CDW_t0_) and glycerol (Gly_t0_) concentrations.; Figure S3: Time-resolved oxygen transfer rate signals of *Hansenula polymorpha* RB11 pC10-*FMD* (P*_FMD_*-GFP) cultivations in SYN6 medium at eight different initial cell dry weights (CDW_t0_) and glycerol (Gly_t0_) concentrations in triplicates.; Figure S4: Individual time-resolved online-monitoring signals of (A) scattered light, (B) GFP fluorescence, (C) score of first (PC1) and (D) second principal component (PC2) based on 2D spectra of *Hansenula polymorpha* RB11 pC10-*FMD* (P*_FMD_*-GFP) cultivations in SYN6 medium at eight different initial cell dry weights (CDW_t0_) and glycerol (Gly_t0_) concentrations.; Figure S5: Score plot of first (PC1) and second (PC2) principal component, based on 2D spectra of *Hansenula polymorpha* RB11 pC10-*FMD* (P*_FMD_*-GFP) cultivations in SYN6 medium at eight different initial cell dry weights (CDWt0) and glycerol (Glyt0) concentrations in sixfold replicates.; Figure S6: Root-mean-square error for (A,D) glycerol, (B,E) cell dry weight and (C,F) pH value for PLS models with one to six LVs, based on 2D spectra of *Hansenula polymorpha* RB11 pC10- *FMD* (P*_FMD_*-GFP) cultivations in SYN 6 medium at different initial cell dry weights and glycerol concentrations.; Figure S7: Results for (A,B) calibration and (C,D) prediction of the duplicate-based PLS models generated for cell dry weight (CDW) including (A,C) two and (B,D) six latent variables and linear interpolation for a 6 h sampling interval, based on 2D spectra of *Hansenula polymorpha* RB11 pC10-*FMD* (P*_FMD_*-GFP) cultivations in SYN6 medium at different initial cell dry weights (CDW_t0_) and glycerol (Gly_t0_) concentrations.; Figure S8: (A–C) Calibration and (D–F) prediction of the PLS model generated by including two latent variables and linear interpolation for a 6 h sampling interval for (A,D) residual glycerol, (B,E) CDW and (C,F) pH value based on averaged 2D spectra.; Figure S9: Parity plots for the average-based PLS prediction of offline parameters (A) glycerol, (B) cell dry weight (CDW) and (C) pH value using two latent variables on averaged 2D spectra from six replicates of *Hansenula polymorpha* RB11 pC10- *FMD* (P*_FMD_*-GFP) cultivations in SYN6 medium at different initial cell dry weights (CDW_t0_) and glycerol (Gly_t0_) concentrations.
