# Supplementary material for: Online 2D Fluorescence Monitoring in Microtiter Plates Allows Prediction of Cultivation Parameters and Considerable Reduction in Sampling Efforts for Parallel Cultivations of Hansenula polymorpha
Source: Bioengineering (Basel). 2022 Sep 4;9(9):438. doi: 10.3390/bioengineering9090438 (PMC9495725; doi:10.3390/bioengineering9090438)
Supplement: Supplementary file 1 [file bioengineering-09-00438-s001.zip › bioengineering-1872767-supplementary.pdf]

Table S1: Absolute and relative RMSE resulting for the PLS model for the glycerol concentration, CDW and pH value based on batch cultures of *Hansenula polymorpha* RB11 pC9-*FMD* (P_{FMD}-GFP) at different initial CDW and glycerol using a variable number of LVs. All models were generated based on the interpolated 6 hs sampling interval. The RMSE of the calibration dataset was calculated using the interpolated offline values of the 6 h or the 1.5 h sampling interval. For the prediction dataset, the respective PLS model was applied and the RMSE was calculated based on the interpolated 1.5 h sampling interval. Further, the RMSE for both the duplicate-based and the sextuplicate average-based approach are shown. Table 1B reproduces the values of Table 1A for the visualized PLS models. Additionally, the relative RMSE was calculated based on the respective offline parameter range and is shown in brackets.

Table S1A

			RMSE duplicate-based model			RMSE six-replicates-average-based model		
		Parameter range	Calibration (6 hours)	Calibration (1.5 hours)	Prediction (1.5 hours)	Calibration (6 hours)	Calibration (1.5 hours)	Prediction (1.5 hours)
Glycerol [g/L]	LV1	0 – 9 g/L	0.61	0.39	0.44	0.33	0.34	0.40
	LV2		0.53	0.31	0.34	0.29	0.29	0.33
	LV3		0.40	0.31	0.58	0.20	0.18	0.27
	LV4		0.38	0.32	0.58	0.18	0.15	0.25
	LV5		0.34	0.33	0.47	0.14	0.16	0.26
	LV6		0.30	0.33	0.45	0.13	0.17	0.27
CDW [g/L]	LV1	0.03 – 3.7 g/L	0.21	0.13	0.15	0.19	0.12	0.15
	LV2		0.19	0.12	0.14	0.18	0.10	0.12
	LV3		0.17	0.13	0.21	0.12	0.12	0.20
	LV4		0.14	0.13	0.24	0.09	0.13	0.17
	LV5		0.13	0.13	0.23	0.08	0.15	0.16
	LV6		0.12	0.12	0.21	0.08	0.15	0.16
pH value [-]	LV1	5.5 – 6.04	0.039	0.030	0.035	0.036	0.028	0.032
	LV2		0.032	0.023	0.029	0.030	0.021	0.028
	LV3		0.025	0.020	0.031	0.019	0.018	0.027
	LV4		0.021	0.020	0.036	0.016	0.020	0.027
	LV5		0.018	0.020	0.032	0.011	0.020	0.029
	LV6		0.016	0.020	0.028	0.009	0.019	0.030

Table S1B

			RMSE duplicate-based model			RMSE six-replicates-average-based model		
		Parameter range	Calibration (6 hours)	Calibration (1.5 hours)	Prediction (1.5 hours)	Calibration (6 hours)	Calibration (1.5 hours)	Prediction (1.5 hours)
Glycerol [g/L]	LV2	0 – 9 g/L	0.53 (5.9 %)	0.31 (3.5 %)	0.34 (3.8 %)	0.29 (3.2 %)	0.29 (3.3 %)	0.33 (3.7 %)
CDW [g/L]	LV2	0.03 – 3.7 g/L	0.19 (5.1 %)	0.12 (3.2 %)	0.14 (3.9 %)	0.18 (4.9 %)	0.10 (2.8 %)	0.12 (3.1 %)
	LV6		0.12 (3.3 %)	0.12 (3.3 %)	0.21 (5.7 %)	0.08 (2.2 %)	0.15 (4.1 %)	0.16 (4.4 %)
pH value [-]	LV2	5.5 – 6.04	0.032 (6.0 %)	0.023 (4.3 %)	0.029 (5.3 %)	0.030 (5.6 %)	0.021 (4.0 %)	0.028 (5.1 %)

Table S2: Overview of the absolute and relative RMSE reported for the PLS models generated for batch cultures of *H. polymorpha* RB11 pC9-*FMD* (P_{FMD}-GFP) at different initial CDW and glycerol concentrations. The values are extracted from this study, as well as from the previous studies by Ladner et al. [45] and Paquet-Durand et al. [46]. The relative RMSE is shown in brackets and was calculated based on the respective offline parameters range.

	This Study		Ladner et al. (2016)		Paquet-Durand et al. (2017)	
	**Calibration** (1.5 hours)	**Prediction** (1.5 hours)	**Calibration** (3 hours)	**Prediction** (3 hours)	**Calibration** (irregular)	**Prediction** (irregular)
**Glycerol [g/L]**	0.31 (3.5 %)	0.34 (3.8 %)	0.18 (1.8 %)	0.26 (2.6 %)	n.a.	1.12 (13.9 %)
**CDW [g/L]**	0.12 (3.2 %)	0.14 (3.9 %)	0.10 (2.3 %)	0.22 (5.2 %)	n.a.	0.19 (4.7 %)
**pH value [-]**	0.023 (4.3 %)	0.029 (5.3 %)	0.022 (4.4 %)	0.035 (7.0 %)	n.a.	n.a.

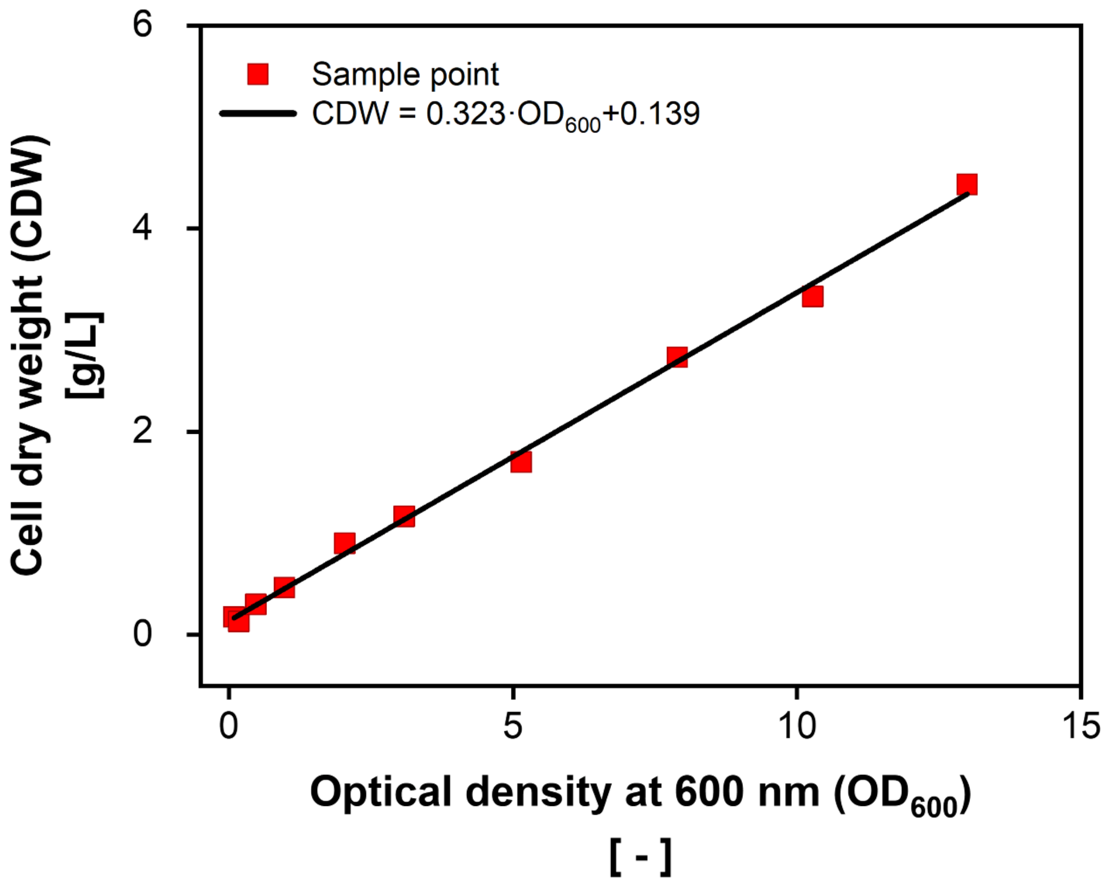

Figure S1: Linear correlation between cell dry weight (CDW) and optical density at 600 nm (OD_{600}). Cultures were grown in SYN6 medium according to the preculture protocol. The shake flask cultures were harvested during exponential growth, before dilution series were prepared with fresh medium. For the OD_{600} measurement of the diluted series, additional dilution was conducted to allow measurement between 0.1 and 0.3 a.u.

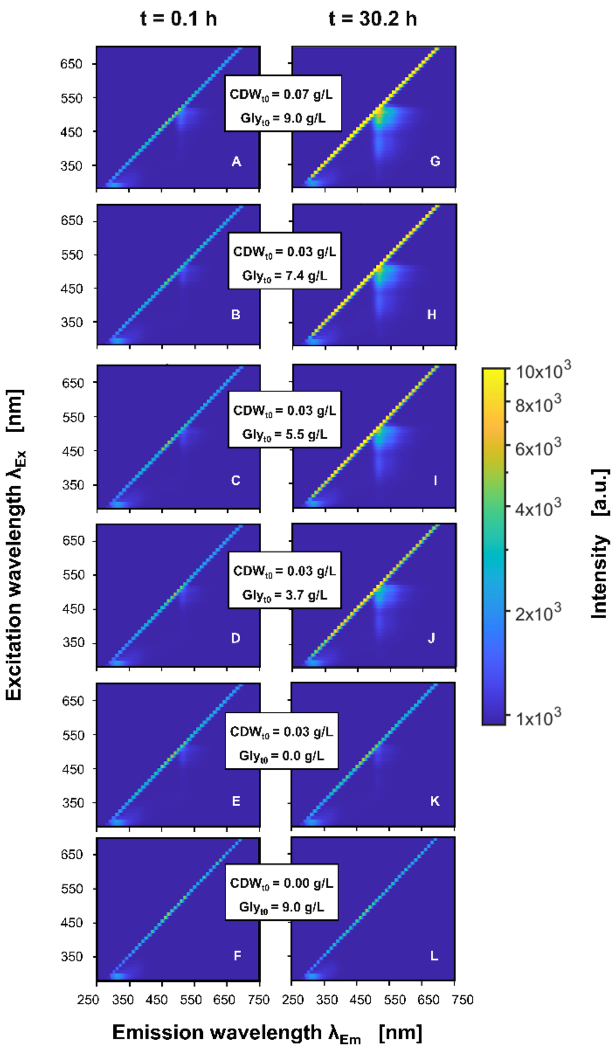

Figure S2: Exemplary 2D spectra of *Hansenula polymorpha* RB11 pC10-*FMD* (P$_{FMD}$-GFP) cultures in SYN6 medium after 0.1 h (A-E) and 30.2 h (G-J) for different initial cell dry weights (CDW$_{t0}$) and glycerol (Gly$_{t0}$) concentrations. The spectra are smoothed according to the methods section, but not referenced to the first cycle. Spectroscopic measurement settings: excitation wavelength range = 280 nm – 700 nm (step size = 10 nm), emission wavelength range = 278 nm – 720 nm (step size = 0.45 nm), Integration time = 30 ms. Cultivation conditions: 48-well microtiter plate with round geometry, liquid volume = 800 µL, shaking diameter = 3 mm, shaking frequency = 1000 rpm, temperature = 30 °C.

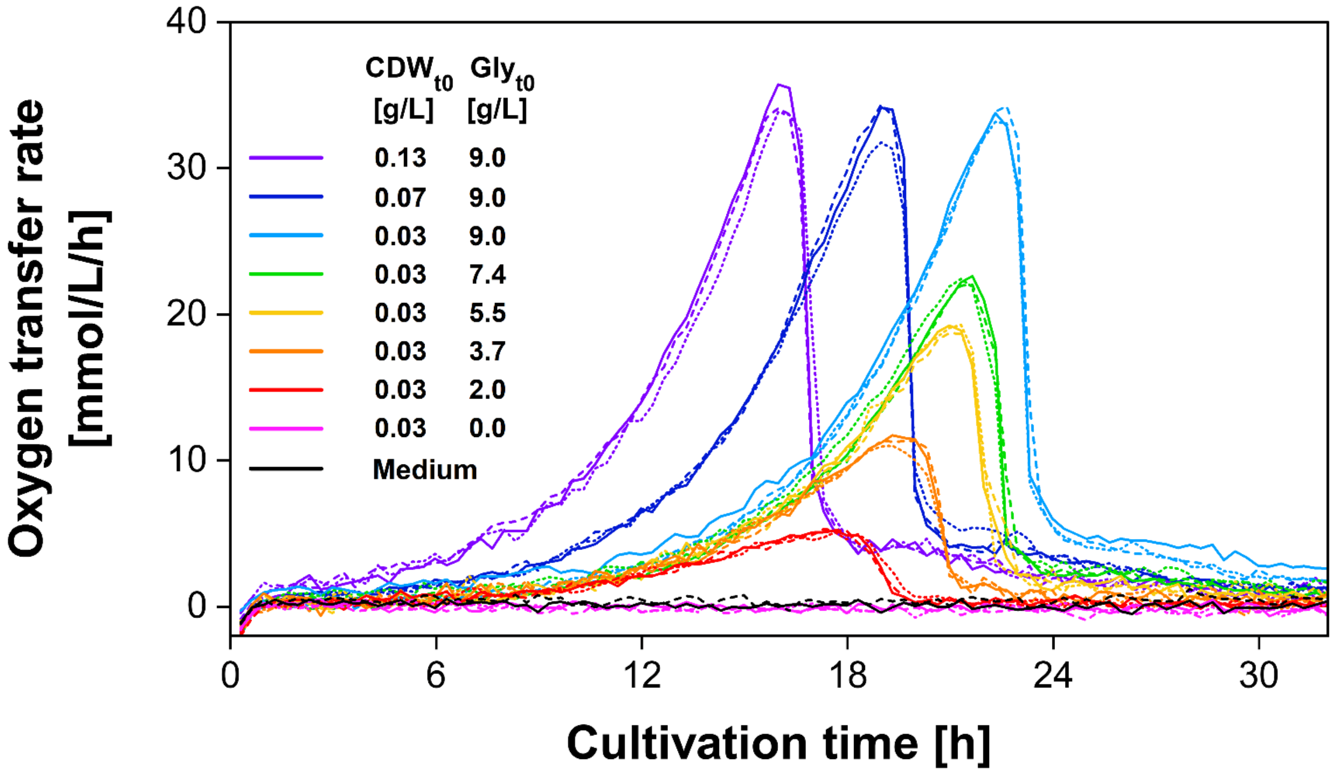

Figure S3: Time-resolved oxygen transfer rate signals of *Hansenula polymorpha* RB11 pC10-*FMD* (P_{FMD}-GFP) cultivations in SYN6 medium at eight different initial cell dry weights (CDW_{t0}) and glycerol (Gly_{t0}) concentrations in triplicates. Solid, dashed, and dotted lines indicate oxygen transfer rates of individual cultures measured in the µRAMOS device. The data was used to calculate the mean and standard deviation of the OTR, as shown in Figure 4A. Cultivation conditions: 48-well microtiter plate with round geometry, liquid volume = 800 µL, shaking diameter = 3 mm, shaking frequency = 1000 rpm, temperature = 30 °C.

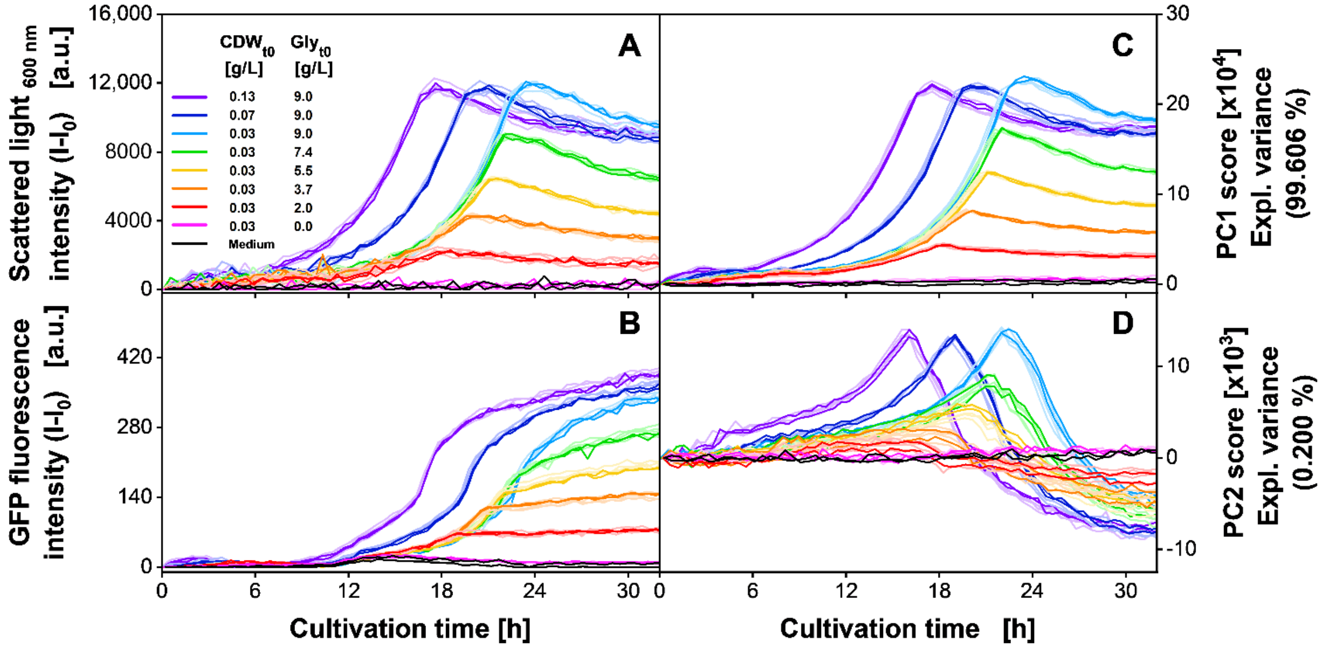

Figure S4: Individual time-resolved online-monitoring signals of (A) scattered light, (B) GFP fluorescence, (C) score of first (PC1) and (D) second principal component (PC2) based on 2D spectra of *Hansenula polymorpha* RB11 pC10-*FMD* (P_{FMD}-GFP) cultivations in SYN6 medium at eight different initial cell dry weights (CDW_{t0}) and glycerol (Gly_{t0}) concentrations. Online signals of single wavelength combinations for six replicates (A-B) indicate a very good well-to-well reproducibility. The online signals are used to calculate the mean and standard deviation shown in Figure 4 B and C. Dark-coloured lines indicate cultures of duplicate wells, used for the calibration of the duplicate based PLS models. Light coloured lines show additional four replicates. The results of the principal component analysis (PCA) (C-D) indicate an explained variance of 99.6% for PC1 and 0.2% explained variance for PC2. Spectroscopic measurement settings: excitation wavelength range = 280 nm – 700 nm (step size = 10 nm), emission wavelength range = 278 nm – 720 nm (step size = 0.45 nm), integration time = 30 ms. Cultivation conditions: 48-well microtiter plate with round geometry, liquid volume = 800 µL, shaking diameter = 3 mm, shaking frequency = 1000 rpm, temperature = 30 °C.

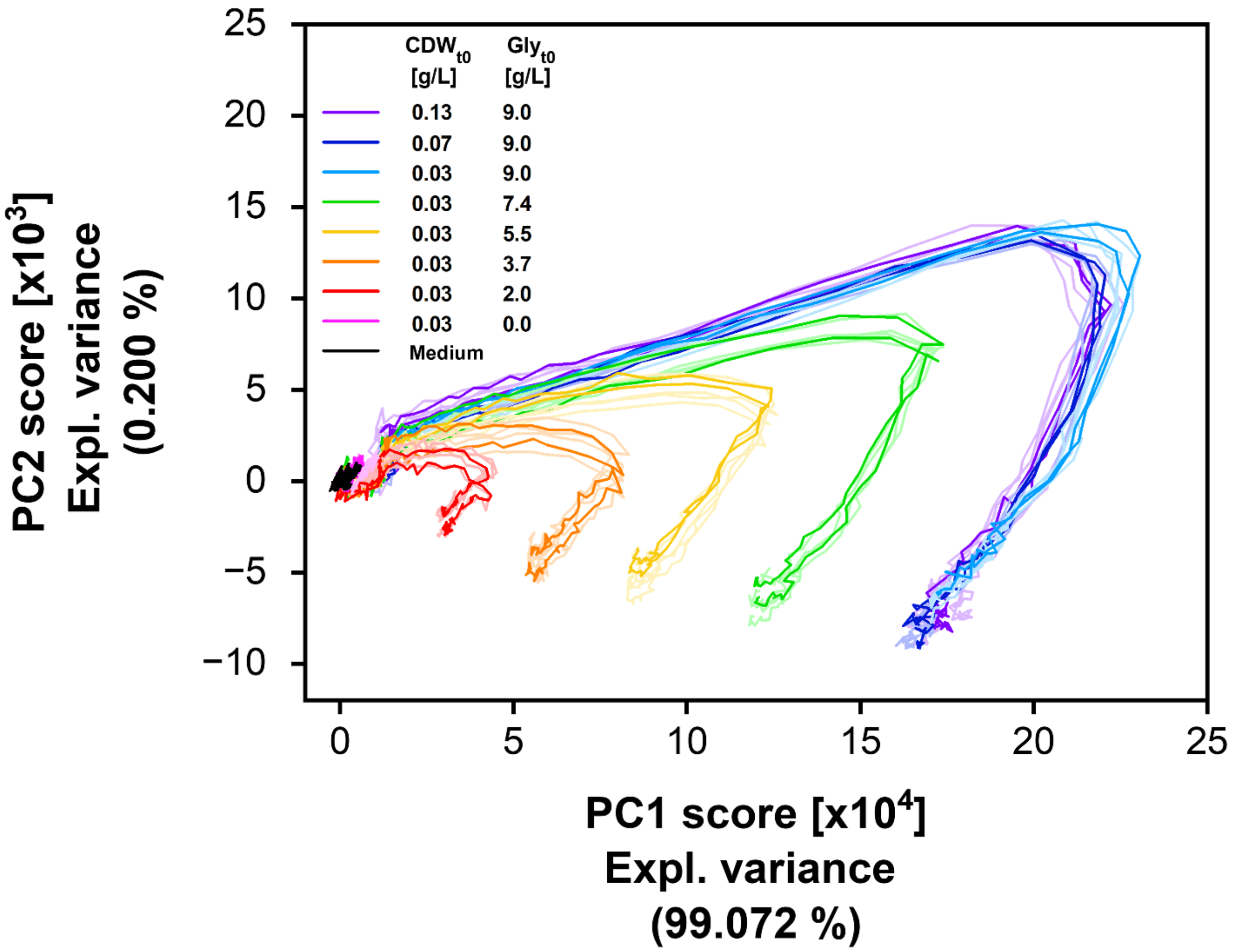

Figure S5: Score plot of first (PC1) and second (PC2) principal component, based on 2D spectra of *Hansenula polymorpha* RB11 pC10-*FMD* (P_{FMD}-GFP) cultivations in SYN6 medium at eight different initial cell dry weights (CDW_{t0}) and glycerol (Gly_{t0}) concentrations in sixfold replicates. Cultures with identical initial glycerol concentrations (purple, blue, light blue) describe identical trajectories. For lower initial glycerol concentrations, the trajectory is flattened as a result from lower PC2 scores. The dark-coloured lines indicate online data chosen for the duplicate-based PLS model. The residual four lines are depicted each in light colours. Spectroscopic measurement settings: excitation wavelength range = 280 nm – 700 nm (step size = 10 nm), emission wavelength range = 278 nm – 720 nm (step size = 0.45 nm), integration time = 30 ms. Cultivation conditions: 48-well microtiter plate with round geometry, liquid volume = 800 µL, shaking diameter = 3 mm, shaking frequency = 1000 rpm, temperature = 30 °C.

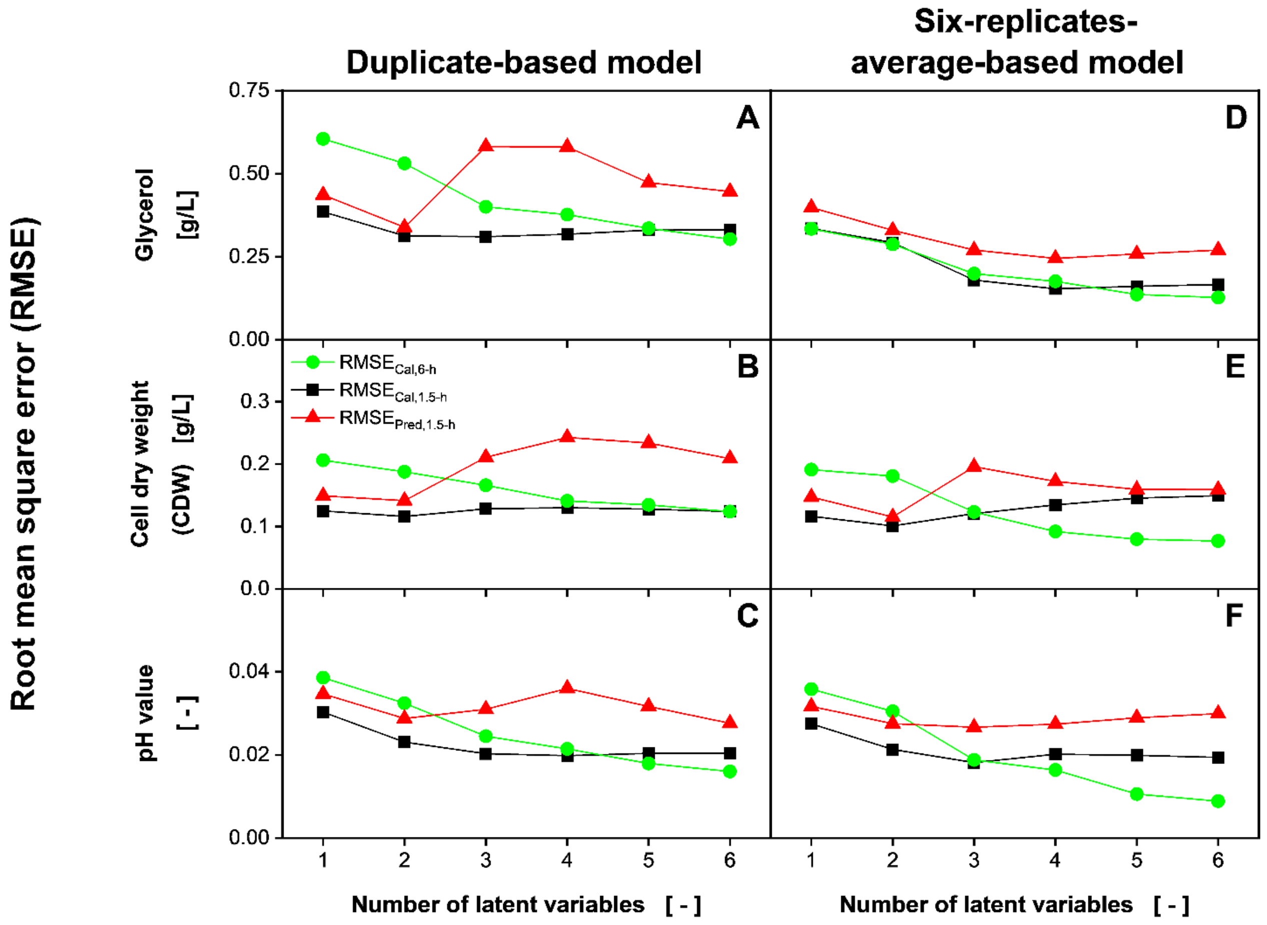

Figure S6: Root mean square error for (A, D) glycerol, (B, E) cell dry weight and (C, F) pH value for PLS models with one to six LVs, based on 2D spectra of *Hansenula polymorpha* RB11 pC10-*FMD* (P_{FMD}-GFP) cultivations in SYN6 medium at different initial cell dry weights (CDW_{t0}) and glycerol concentrations. For all PLS models the calibration dataset consisted of the 6 h sampling interval. The RMSE is calculated based on the linear interpolation of the 6 h ($RMSE_{Cal,6\text{-}h}$, green circles) and the 1.5 h sampling interval ($RMSE_{Cal,1.5\text{-}h}$, black squares). For the prediction dataset, the RMSE resulting from the application of the respective PLS model is calculated only for the 1.5 h sampling interval ($RMSE_{Pred,1.5\text{-}h}$, red upwards triangles). Resulting errors are shown for both the duplicate-based (A-C) and the six replicates average-based model (D-F).

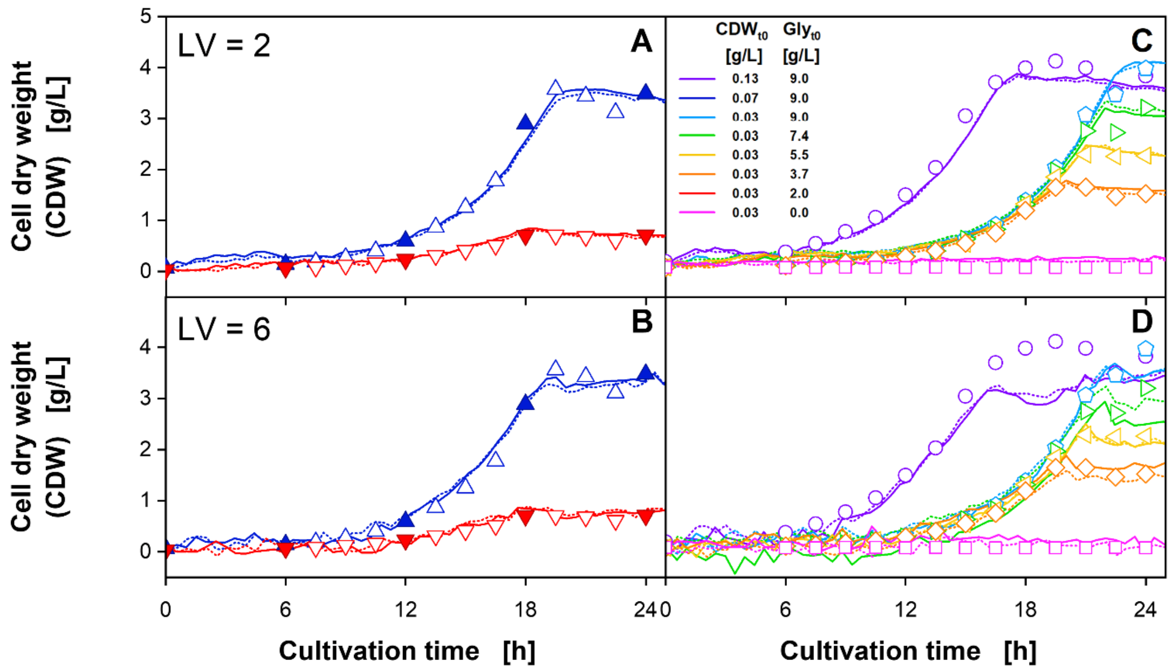

Figure S7: Results for (A-B) calibration and (C-D) prediction of the duplicate-based PLS models generated for cell dry weight (CDW) including (A, C) two and (B, D) six latent variables and linear interpolation for a 6 h sampling interval, based on 2D spectra of *Hansenula polymorpha* RB11 pC10-*FMD* (P_{FMD}-GFP) cultivations in SYN6 medium at different initial cell dry weights (CDW_{t0}) and glycerol (Gly_{t0}) concentrations. For calibration, a total of 212 2D spectra from duplicates of *Hansenula polymorpha* RB11 pC10-FMD (P_{FMD}-GFP) cultivations for initial cell dry weights (CDW_{t0}) and glycerol concentration (Gly_{t0}) of 0.07 g/L and 9.0 g/L (blue) as well as of 0.03 g/L and 2.0 g/L (red) were included. The offline values used for linear interpolation and subsequent calibration are shown as filled symbols. Hollow symbols are shown for validation only. The solid and dotted lines describe duplicates as indicated in Supplementary Figure 3. Cultivation conditions: 48-well microtiter plate with round geometry, liquid volume = 800 µL, shaking diameter = 3 mm, shaking frequency = 1000 rpm, temperature = 30 °C.

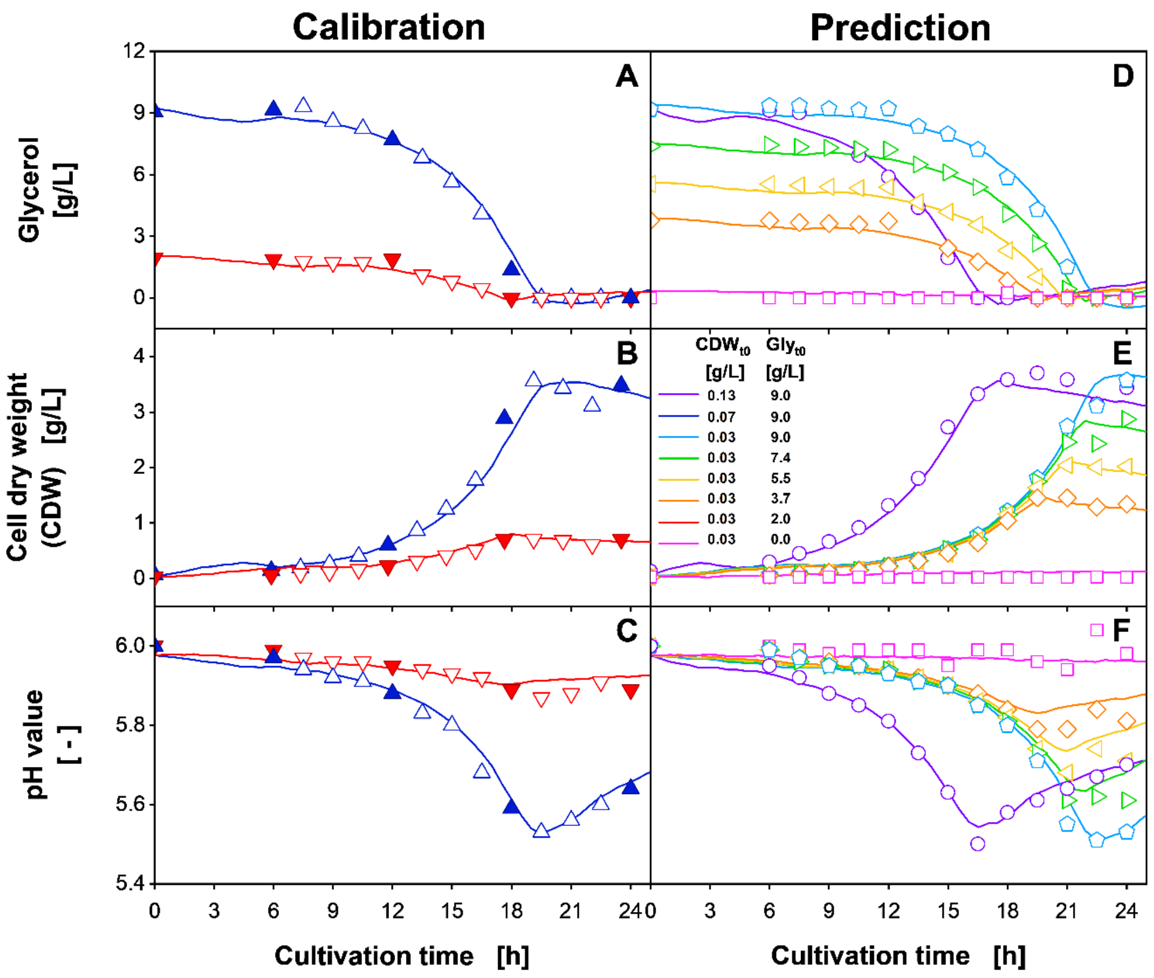

Figure S8: (A-C) Calibration and (D-F) prediction of the PLS model generated by including two latent variables and linear interpolation for a 6 h sampling interval for (A, D) residual glycerol, (B, E) CDW and (C, F) pH value based on averaged 2D spectra. For calibration, 106 2D spectra of *Hansenula polymorpha* RB11 pC10-*FMD* (P_{FMD}-GFP) cultivations with an initial cell dry weight (CDW_{t0}) and a glycerol concentration (Gly_{t0}) of 0.07 g/L and 9.0 g/L (blue) as well as of 0.03 g/L and 2.0 g/L (red) were calculated by averaging the spectral data of all six replicates according to initial cultivation conditions and cultivation time. In total, 636 2D spectra were included to calculate 106 average spectra used for PLS model calibration. The offline values used for linear interpolation and subsequent calibration are shown as filled symbols and are identical dal the data shown in Figure 5. Hollow symbols are shown for validation purposes. Cultivation conditions: 48-well microtiter plate with round geometry, liquid volume = 800 µL, shaking diameter = 3 mm, shaking frequency = 1000 rpm, temperature = 30 °C.

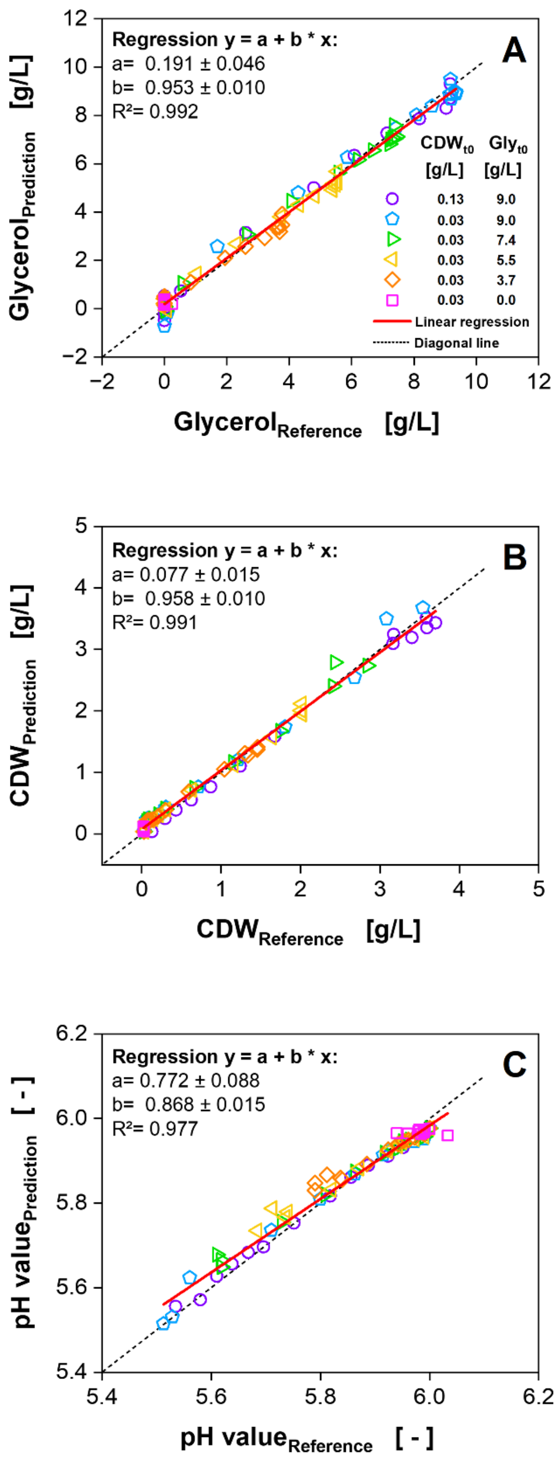

Figure S9: Parity plots for the average-based PLS prediction of offline parameters (A) glycerol, (B) cell dry weight (CDW) and (C) pH value using two latent variables on averaged 2D spectra from six replicates of *Hansenula polymorpha* RB11 pC10-*FMD* (P_{FMD}-GFP) cultivations in SYN6 medium at different initial cell dry weights (CDW_{t0}) and glycerol (Gly_{t0}) concentrations. Only data points with corresponding offline sampling data, as shown by a hollow symbol in Figure 6, are plotted. Red line indicates the resulting linear regression of predicted values and reference offline values.
