# Supplementary figures and images for: Online 2D Fluorescence Monitoring in Microtiter Plates Allows Prediction of Cultivation Parameters and Considerable Reduction in Sampling Efforts for Parallel Cultivations of Hansenula polymorpha
Source: Bioengineering (Basel). 2022 Sep 4;9(9):438. doi: 10.3390/bioengineering9090438 (PMC9495725; doi:10.3390/bioengineering9090438)

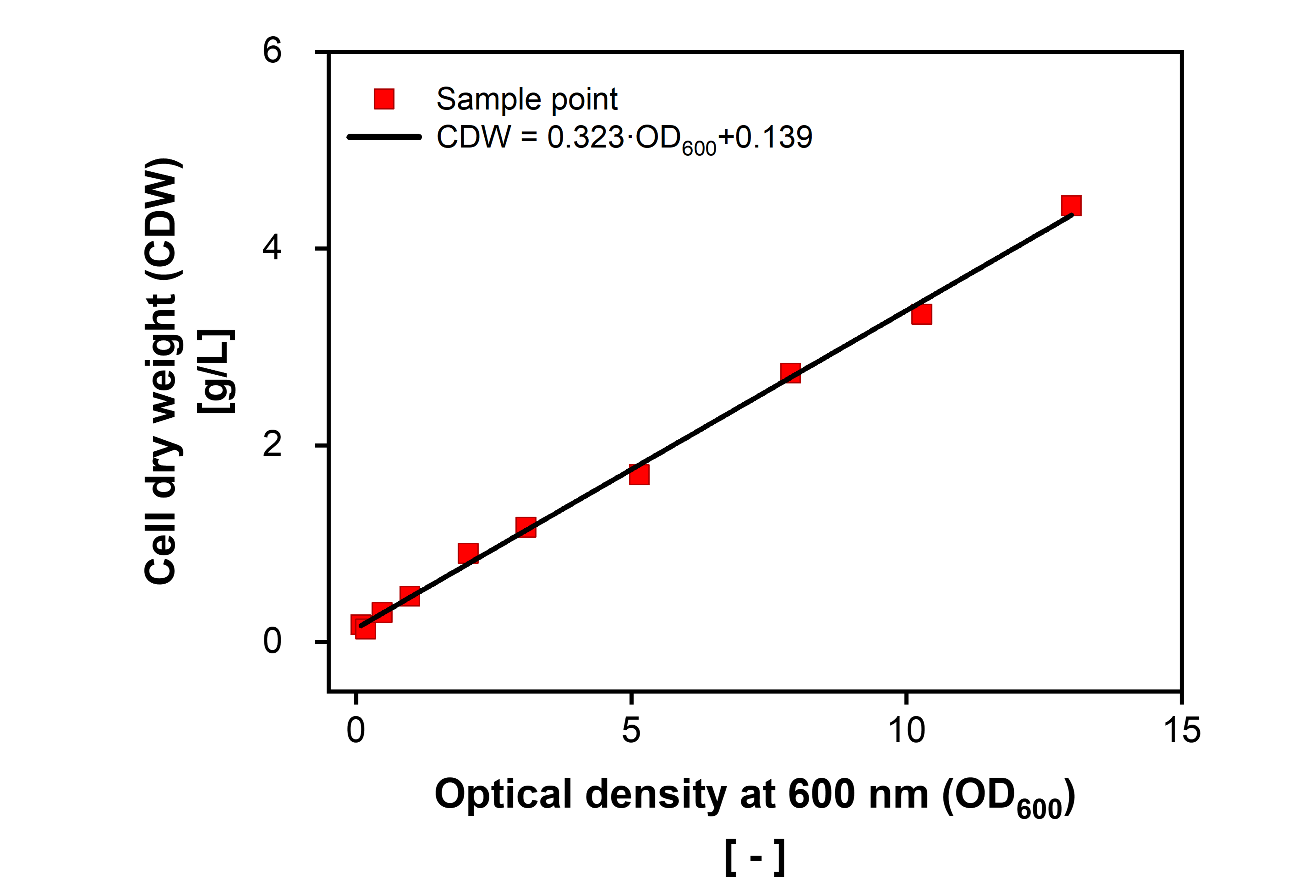

Supplement: Supplementary file 1 [file bioengineering-09-00438-s001.zip › SuppS1.tif]

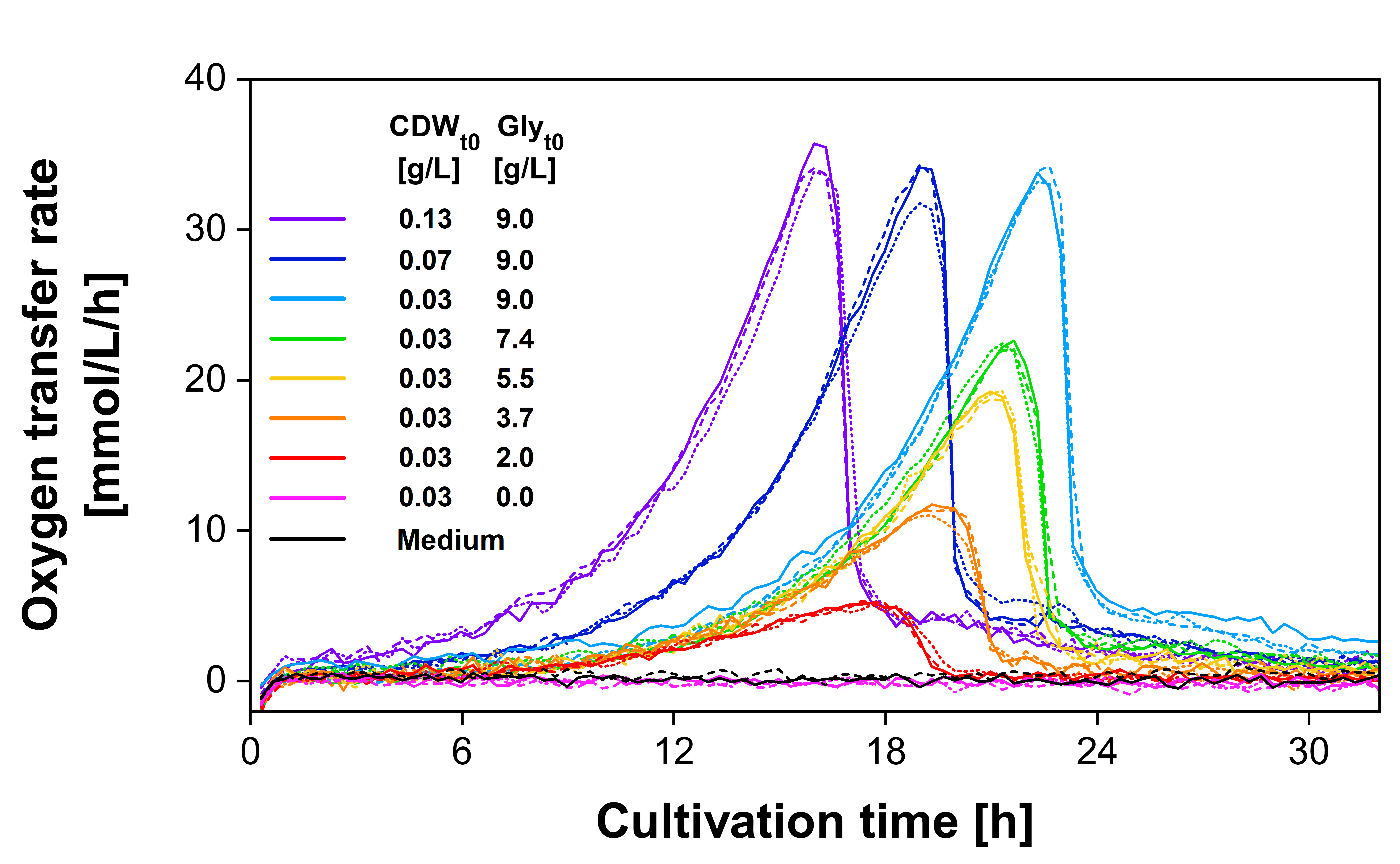

Supplement: Supplementary file 1 [file bioengineering-09-00438-s001.zip › SuppS3.TIF]

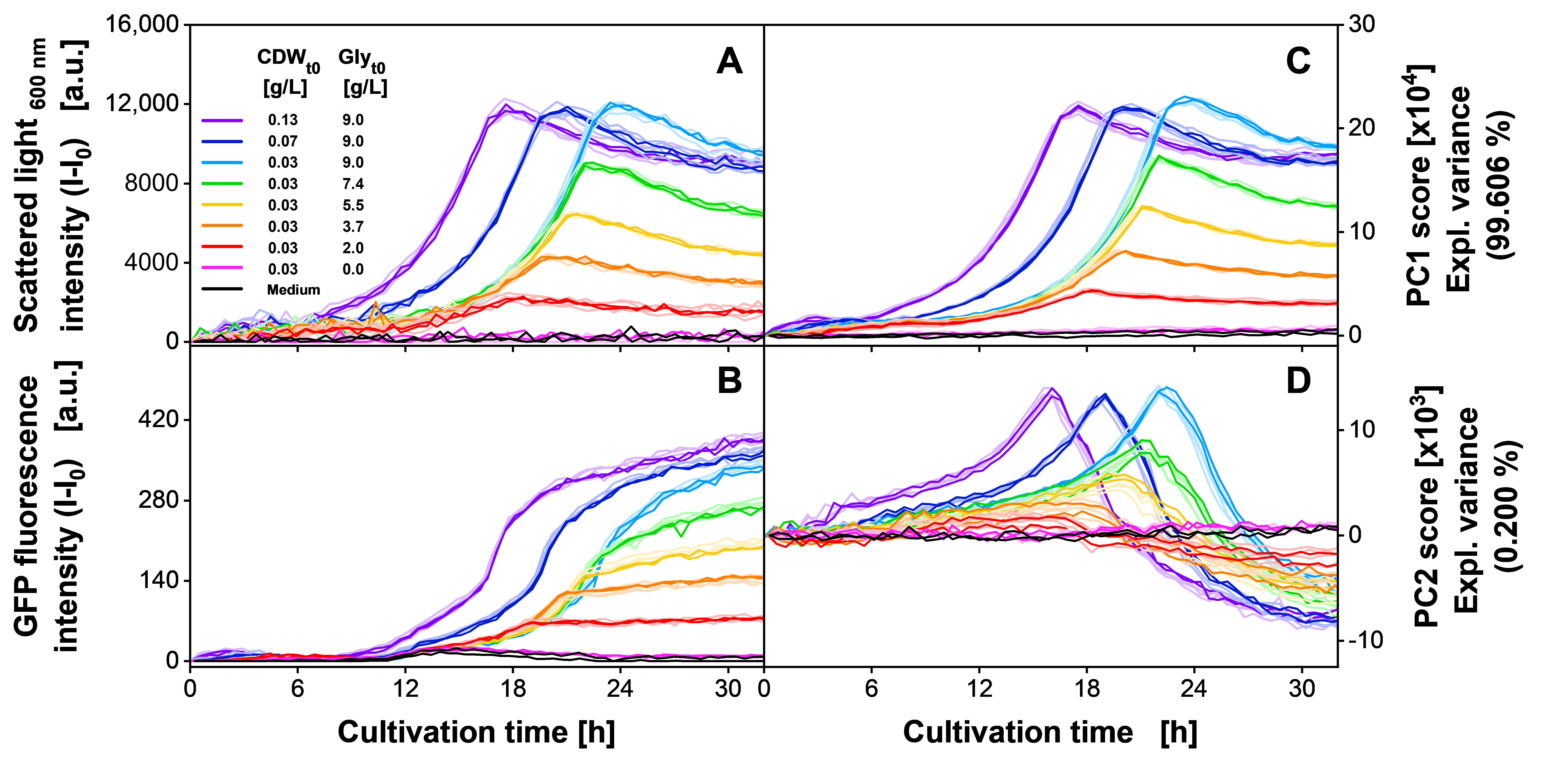

Supplement: Supplementary file 1 [file bioengineering-09-00438-s001.zip › SuppS4.tif]

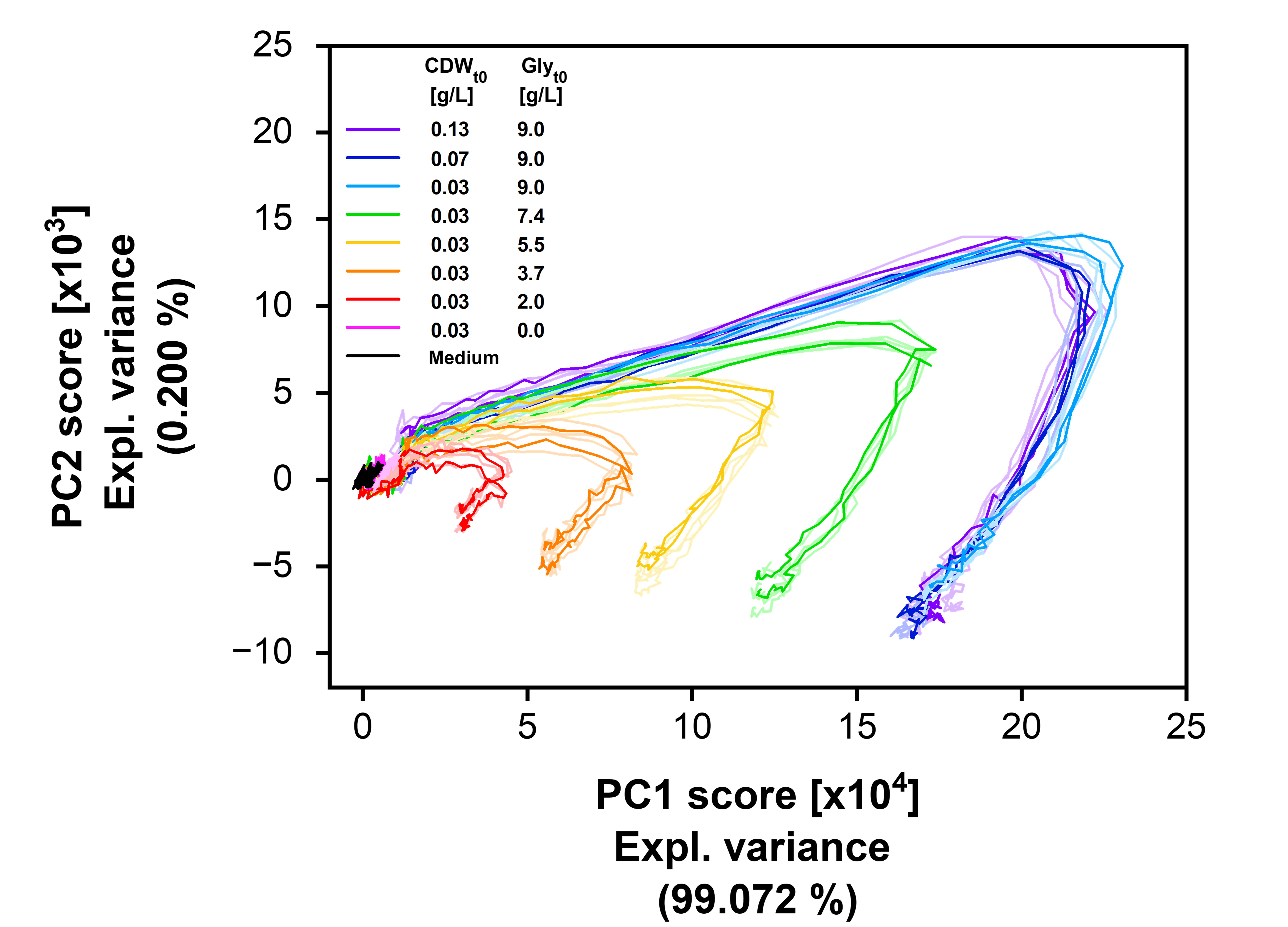

Supplement: Supplementary file 1 [file bioengineering-09-00438-s001.zip › SuppS5.tif]

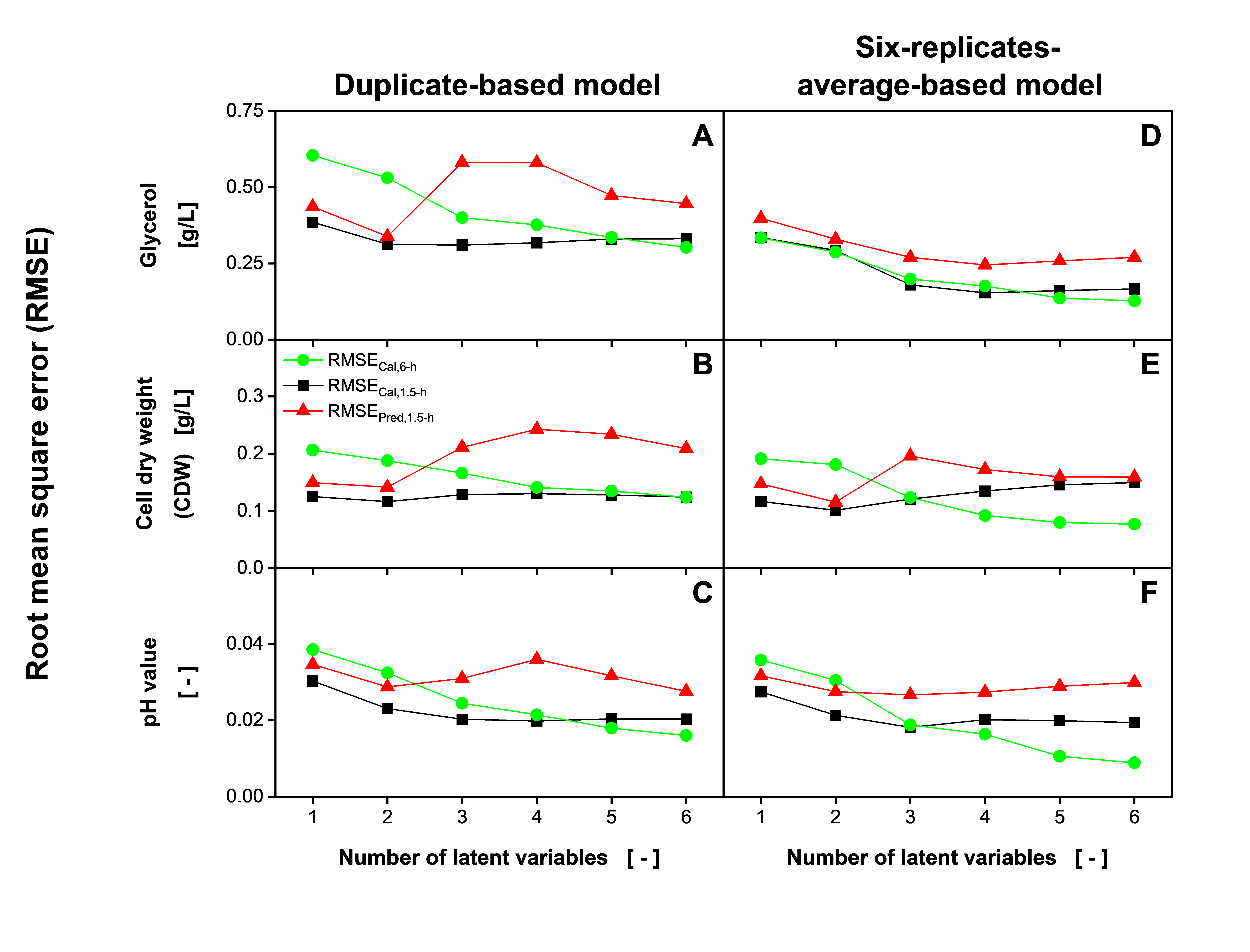

Supplement: Supplementary file 1 [file bioengineering-09-00438-s001.zip › SuppS6.tif]

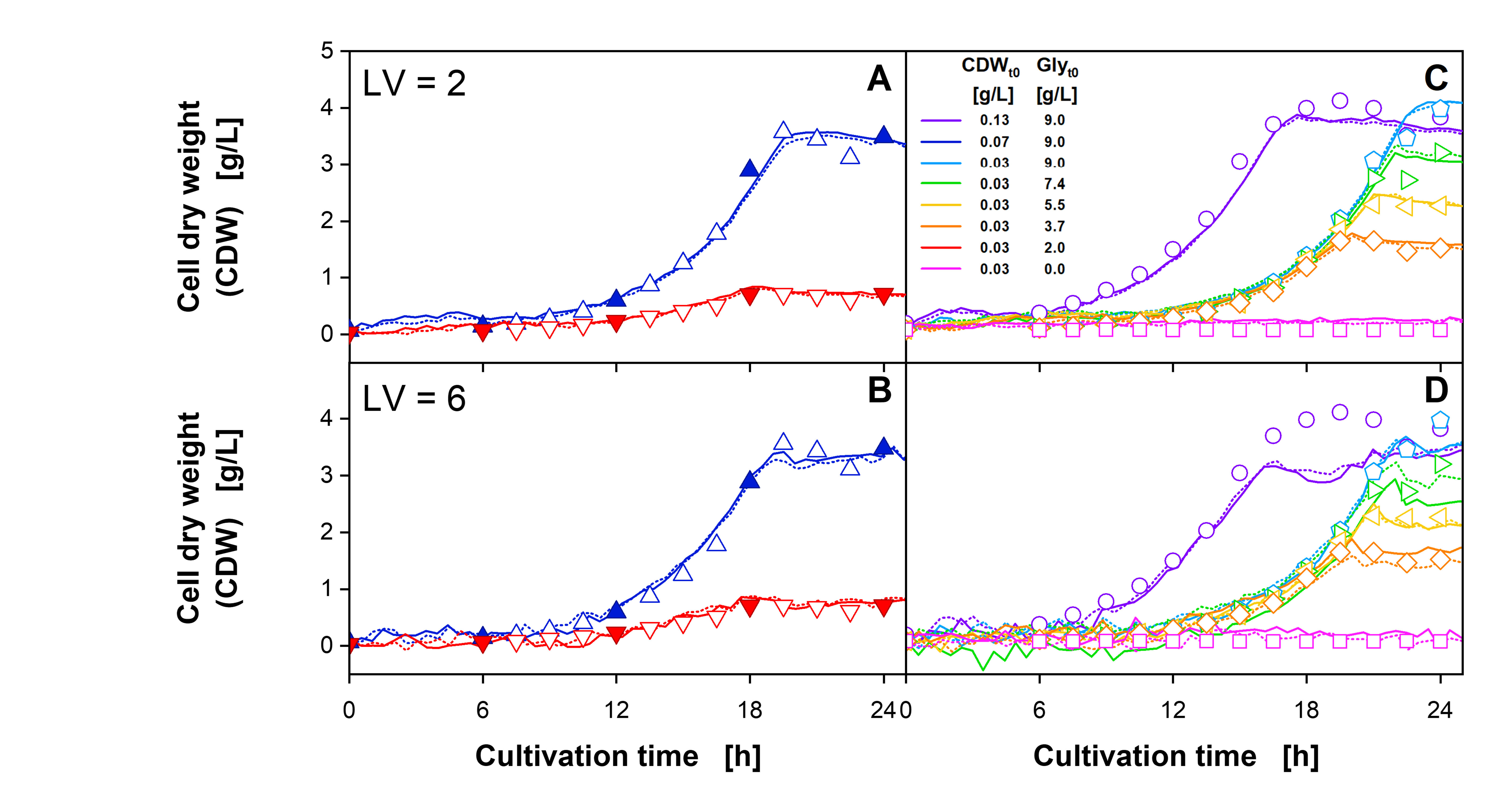

Supplement: Supplementary file 1 [file bioengineering-09-00438-s001.zip › SuppS7.tif]

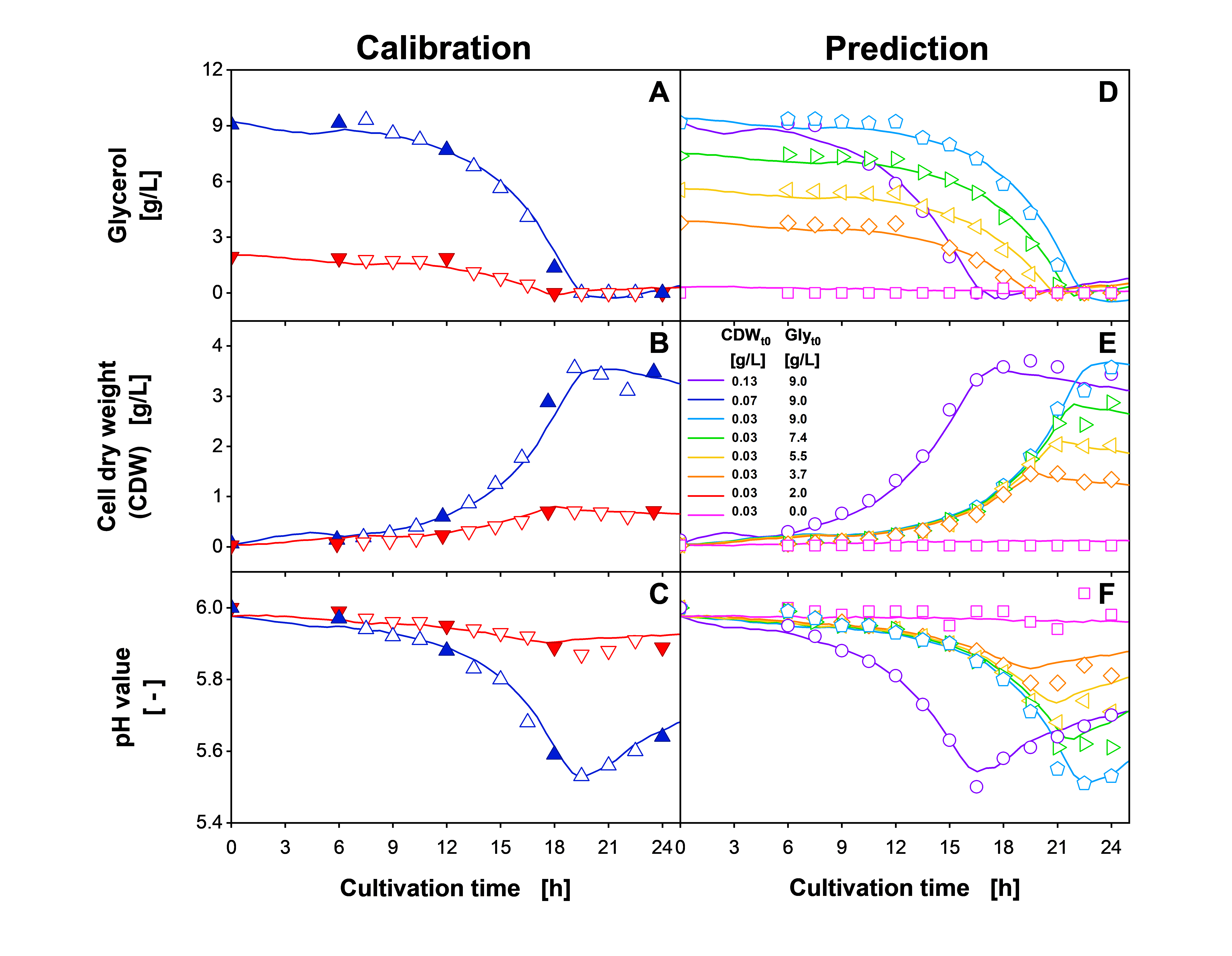

Supplement: Supplementary file 1 [file bioengineering-09-00438-s001.zip › SuppS8.tif]

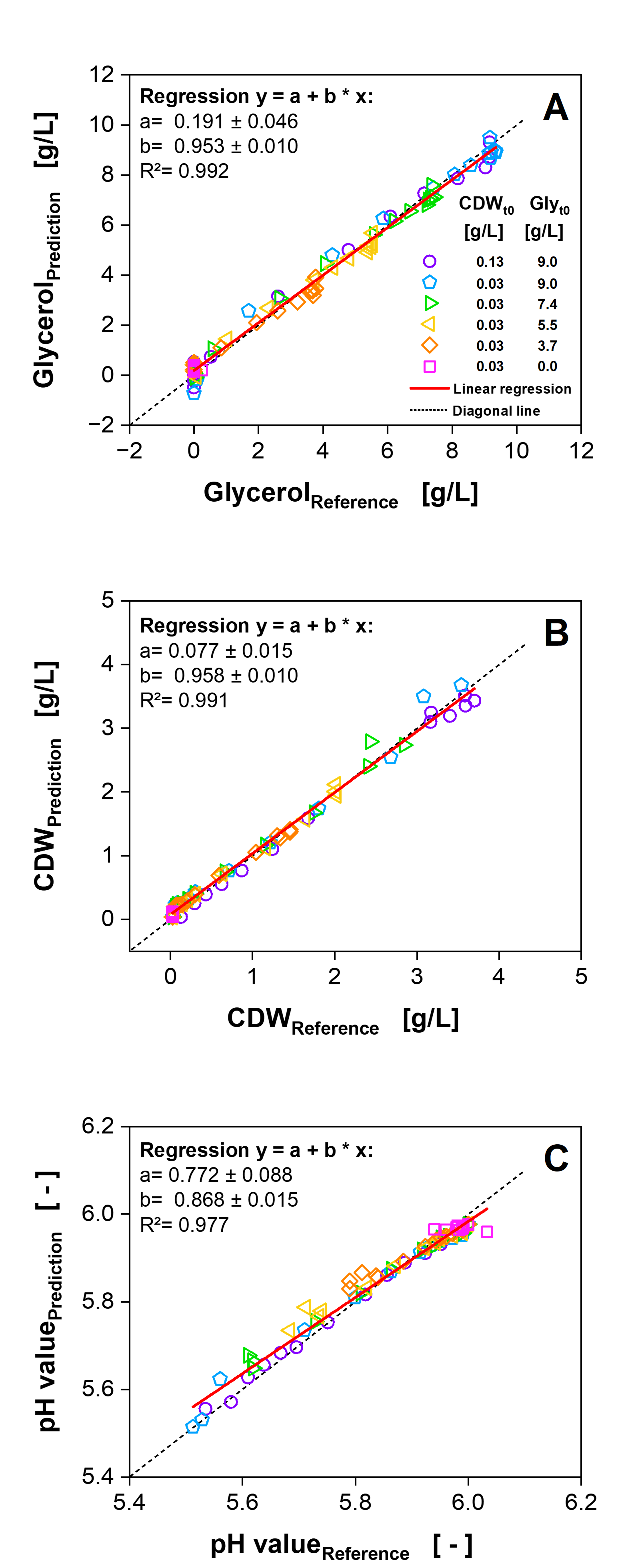

Supplement: Supplementary file 1 [file bioengineering-09-00438-s001.zip › SuppS9.tif]
